# Pinpointing top inhibitors for GSK3β from pool of indirubin derivatives using rigorous computational workflow and their validation using molecular dynamics (MD) simulations

**DOI:** 10.1038/s41598-023-50992-7

**Published:** 2024-01-02

**Authors:** Vamangi Pandya, Priyashi Rao, Jignesh Prajapati, Rakesh M. Rawal, Dweipayan Goswami

**Affiliations:** 1 L. J. School of Applied Sciences, L. J. University, Sarkhej, Ahmedabad, 380051 India; 2 Department of Biochemistry and Forensic Science, University School of Sciences, Gujarat University, Ahmedabad, Gujarat 380009 India; 3 Department of Life Science, University School of Sciences, Gujarat University, Ahmedabad, Gujarat 380009 India; 4 Department of Microbiology and Biotechnology, University School of Sciences, Gujarat University, Ahmedabad, Gujarat 380009 India

**Keywords:** Computational biology and bioinformatics, Drug discovery

## Abstract

Glycogen synthase kinase-3β (GSK3β) is a pivotal protein kinase implicated in a spectrum of debilitating diseases, encompassing cancer, diabetes, and neurodegenerative disorders. While the therapeutic potential of GSK3β inhibition is widely recognized, there remains an unmet need for a rigorous, systematic analysis probing the theoretical inhibition dynamics of a comprehensive library of indirubin derivatives against GSK3β using advanced computational methodologies. Addressing this gap, this study embarked on an ambitious endeavor, leveraging indirubin—a renowned scaffold—as a template to curate a vast library of 1000 indirubin derivatives from PubChem. These were enriched with varied substitutions and modifications, identified via a structure similarity search with a Tanimoto similarity threshold of 85%. Harnessing a robust virtual screening workflow, we meticulously identified the top 10 contenders based on XP docking scores. Delving deeper, we gauged the binding free energy differentials (ΔGBind) of these hits, spotlighting the top three compounds that showcased unparalleled binding prowess. A comparative pharmacophore feature mapping with the reference inhibitor OH8, co-crystallized with GSK3β (PDB ID: 6Y9R), was undertaken. The binding dynamics of these elite compounds were further corroborated with 100 ns molecular dynamics simulations, underlining their stable and potent interactions with GSK3β. Remarkably, our findings unveil that these indirubin derivatives not only match but, in certain scenarios, surpass the binding affinity and specificity of OH8. By bridging this research chasm, our study amplifies the therapeutic promise of indirubin derivatives, positioning them as frontrunners in the quest for groundbreaking GSK3β inhibitors, potentially revolutionizing treatments for a myriad of ailments.

## Introduction

Glycogen synthase kinase 3 (GSK3) is a family of enzymes that add phosphate groups to other proteins on serine or threonine residues. In mammals, there are two forms of GSK3, GSK3α and GSK3β, which are produced by two separate genes. GSK3α and GSK3β share a high degree of similarity (~ 98%) in their kinase domain and have many overlapping functions, but they also have some unique characteristics and roles^1^. GSK3α and GSK3β participate in various cellular processes, such as glycogen metabolism, gene transcription, apoptosis, and microtubule stability. GSK3α and GSK3β are normally active in quiescent cells and are controlled by inhibition or redirection of their activity by various factors, such as hormones, growth factors, and signaling pathways. GSK3β is a highly connected enzyme, which regulates a multitude of physiological functions in peripheral tissues and the central nervous system (CNS), ranging from metabolism and cell cycle regulation to brain development^2^. Therefore, balanced GSK3β regulation and signaling is essential for human physiology but often impaired in various disease conditions such as diabetes, cancers, and brain disorders^3^. For CNS-related pathologies, abnormal GSK3β activity has been observed in neurological or neurodevelopmental diseases, such as neurodegenerative disorders (Alzheimer’s and Parkinson’s diseases), bipolar disorder, depression, and Fragile X syndrome. Furthermore, clinical, genetic, and pharmacological studies suggested that GSK3β inhibition may improve signaling dysfunction in such diseases. In fact, the role of GSK3β in mood disorder was revealed by studies on the mechanism of actions of the established treatments lithium and valproate, which both inhibited GSK3β^4^. Based on this evidence, extensive efforts have been made in the search for novel GSK3β inhibitors as innovative therapeutic agents, which, however, have not been applied in clinical practice yet. Therefore, GSK3β inhibitors are still required^1,5^.

Indirubin derivatives, encompassing a variety of molecules based on the structure of indirubin, a naturally occurring pigment, exhibit diverse biological activities and potential therapeutic applications. Indirubin derivatives have been shown to have anti-inflammatory, anti-cancer, and neuroprotective effects by inhibiting various protein kinases, such as CDKs and GSK3^6,7^. One of the mechanisms by which indirubin derivatives interact with GSK3 is by competing with adenosine triphosphate (ATP) for the binding site on the kinase domain. This prevents GSK3 from phosphorylating its substrates, such as glycogen synthase, β-catenin, and tau^8,9^. By inhibiting GSK3, indirubin derivatives can modulate various signaling pathways that are involved in inflammation, cancer, and brain disorders. For example, indirubin derivatives can suppress the activation of NF-κB and STAT3, which are transcription factors that regulate the expression of genes related to inflammation, angiogenesis, and tumorigenesis^8,10^. Indirubin derivatives can also enhance the stability of β-catenin, which is a key component of the Wnt signaling pathway that regulates cell proliferation, differentiation, and survival. Furthermore, indirubin derivatives can reduce the hyperphosphorylation of tau, which is a microtubule-associated protein that is implicated in neurodegenerative diseases such as Alzheimer’s and Parkinson’s^8,10^. Indirubin derivatives have different structures and properties that affect their selectivity and potency toward GSK3 and other kinases. Some of the factors that influence the interaction of indirubin derivatives with GSK3 are the position and nature of the substituents on the indirubin core, the conformation and orientation of the indirubin ring system, and the hydrogen bonding and hydrophobic interactions with the amino acid residues in the ATP-binding pocket^6,8,9^. Therefore, indirubin derivatives can be designed and optimized to achieve specific therapeutic effects by targeting GSK3 and other kinases.

A significant gap in present-day research is the lack of a thorough and systematic exploration into how indirubin derivatives theoretically inhibit GSK3β, which has not been extensively studied using molecular docking and molecular dynamics (MD) simulations. This approach could facilitate the discovery of the most potent and selective indirubin derivatives for GSK3β inhibition, as well as reveal the key structural and energetic factors that influence their binding affinity and specificity. Moreover, this approach could provide insights into the conformational changes and interactions of GSK3β and its inhibitors, which could assist the rational design and optimization of novel indirubin derivatives as cutting-edge therapeutic agents for various diseases involving GSK3β dysfunction. Therefore, a comprehensive and meticulous study is required to predict the inhibition potential of GSK3β by a diverse array of indirubin derivatives through molecular docking and MD simulations. This approach would facilitate the selection of optimal candidates and disseminate these findings to the research community for further exploration. The scope and power of employing computational in silico studies in this context are multifaceted. These studies allow for a rapid and cost-effective evaluation of numerous compounds, enabling the identification of promising leads with high efficiency^11–13^. Additionally, computational methods offer the advantage of analysing and predicting the molecular basis of protein–ligand interactions, which can facilitate the rational design and optimization of targeted therapeutics^14^.

**Figure 1:**
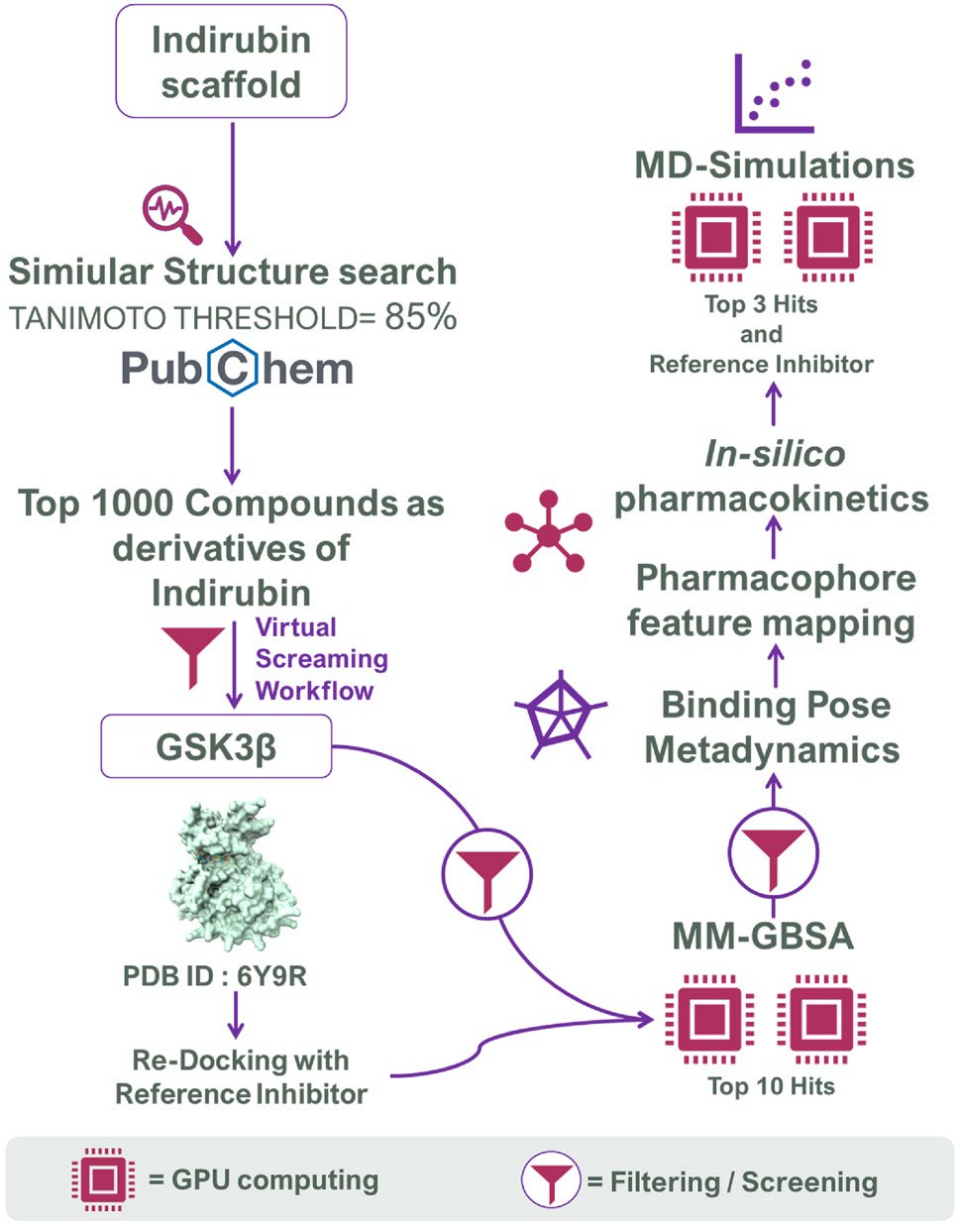
Flow chart of the tasks employed in the current research.

To address this research gap, we conducted an in-depth characterization of the indirubin scaffold to identify and generate a large library of indirubin derivatives possessing various substitutions and modifications from PubChem^15–17^ using a Tanimoto similarity threshold of 85% to select the most similar compounds. This resulted in a manually curated library of 1000 indirubin derivatives, which we subjected to a virtual screening workflow (consisting of high-throughput virtual screening (HTVS), standard precision (SP) and extra precision (XP)) to obtain the top 10 hits based on XP docking scores. We then evaluated the molecular mechanics generalized Born surface area (MM-GBSA) scores of these hits and ranked the top 3 compounds with the lowest binding free energy difference (ΔGBind) energies. We further assessed the pharmacophore feature mapping of these compounds with the reference inhibitor OH8, which was co-crystallized with the GSK3β (PDB ID: 6Y9R) used under this study. We also validated their binding dynamics using molecular dynamics (MD) simulations of 100 ns. By performing this study, we aim to address a significant research gap by providing a comprehensive and systematic analysis of the theoretical inhibition of GSK3β using a large library of indirubin derivatives. The workflow of the current study is represented in Fig. 1. Thus, our study has the potential to advance knowledge in the field and contribute to the discovery of novel GSK3β inhibitors.

## Materials and method

### Ligand library preparation

To curate a focused library of indirubin derivatives for this investigation, we initiated a structure similarity search within the PubChem database^15–17^, utilizing indirubin as the reference molecule for compound identification. The search criteria were anchored on the Tanimoto coefficient, a measure of chemical similarity, with a threshold set at ≥ 0.85 to ensure a high degree of structural resemblance to the indirubin scaffold. This stringent threshold was deliberately chosen to maintain structural integrity, and upon application, yielded an initial list of compounds exceeding our target number. For practical considerations related to computational and experimental manageability, we constrained our selection to the first 1000 compounds ranking highest in similarity as determined by their Tanimoto scores. It is important to note that while a Tanimoto threshold of 0.86 identified 917 compounds, our objective was to examine a library of precisely 1000 compounds. Thus, we adjusted the threshold marginally to 0.85 to meet our numerical goal. This approach assured us of a substantial set of molecules strictly aligned with the structural framework of indirubin, without imposing additional selection biases based on other properties such as bioactivity or physicochemical attributes. Before docking calculations, the identified compounds were prepared using the LigPrep module in Schrödinger Maestro^18^. LigPrep pre-processes ligand structures by generating tautomers, ionization states, and stereochemistries. In this study, ionization states were generated at pH 7.0 ± 2.0 using the Epik module^19^. Tautomers were generated with the default settings, and a maximum of 32 stereoisomers per ligand was allowed. The 3D structures of the ligands were then energy-minimized using the OPLS3e force field^20^ to ensure that they adopt low-energy conformations before docking calculations. The minimized structures were saved in the appropriate format for subsequent docking and molecular dynamics simulations.

### Protein structure preparation of GSK3β and redocking of OH8

We obtained the X-ray crystal structure of GSK3β in complex with reference inhibitor 1H-indazole-3-carboxamide inhibitor 2 (OH8) (PDB ID: 6Y9R) from the Protein Data Bank^21^. This structure had only one chain, ‘Chain A’, which corresponded to GSK3β. We considered the active site of OH8 within Chain A for the docking studies. We prepared the protein structure using the Protein Preparation Wizard module in Schrödinger Maestro^18^, which involved adding hydrogen atoms, removing water molecules, and optimizing hydrogen bond assignments. We then minimized the structure using the OPLS3e force field^20^ to optimize the geometry. To validate the docking protocol and evaluate the chosen method’s ability to reproduce the experimental binding mode of OH8, we performed a redocking procedure. For redocking, we retrieved OH8 from PubChem (CID: 146036038) in SDF format and prepared it using the LigPrep module in Schrödinger Maestro^18^. We then redocked the prepared ligand into the active site (at the same co-ordinates of co-crystalized OH8) of the prepared protein structure using the XP docking module of Glide in Schrödinger Maestro^18^, and compared the resulting binding mode to the crystallographic pose. A successful redocking would result in a low root-mean-square deviation (RMSD) between the redocked pose and the experimental binding mode, indicating that the docking protocol is suitable for predicting the binding modes of the indirubin derivatives in subsequent steps.

### Virtual screening workflow to screen library of indirubin derivatives

To identify potential indirubin derivatives as inhibitors of GSK3β, molecular docking was performed on the 1000 prepared compounds using the Glide module in Schrödinger Maestro^18^. Docking was carried out at the same binding site where OH8 interacts with GSK3β, ensuring that the derivatives were evaluated for their potential to bind at the same site and inhibit GSK3β activity. The binding pocket of GSK3β was defined as a sphere with a radius of 10 Å centered on the co-crystallized OH8 molecule. The binding pocket residues include Ala170, His173, Ser174, Gly176, Leu207, Val208, Arg209, Gly210, Glu211, Asn213, Glu226, Ala231, Thr232, Asp233, Tyr234, Thr235, Ser236, Ser237, Val240, Glu283, Met284, Asn285, Thr324, Pro325, Thr326, Arg328, Leu329, Thr330, Pro331, Leu332, Glu366, Ser368 and Ser369. The area of the binding pocket was estimated to be 449.991 (SA) Å^2^ and volume to be 502.806 (SA) Å^3^ which was determined using CASTp 3.0^22^. These residues are involved in various interactions with OH8 and other GSK3β inhibitors.

The molecular docking process was performed in a hierarchical manner, utilizing three levels of precision: High-throughput Virtual Screening (HTVS), Standard Precision (SP), and Extra Precision (XP). This approach allows for the efficient screening of large compound libraries by first applying a less computationally demanding method (HTVS) to rapidly filter out compounds with low binding potential, followed by more accurate and computationally intensive methods (SP and XP) to refine the ranking and poses of the remaining compounds^23–25^. Initially, all 1000 compounds were docked using the HTVS protocol, and the top-ranked compounds were selected based on their Glide docking scores. These selected compounds were then subjected to the more accurate SP docking protocol, and the top-ranked compounds from this stage were further refined using the XP docking protocol. The final stage involved XP docking, which is the most accurate and computationally demanding docking protocol available in Glide. XP docking employs advanced scoring functions and optimization algorithms to provide more accurate binding pose predictions and refined scoring. Finally, the top 10 compounds with the highest XP docking scores were identified as potential GSK3β inhibitors and considered for subsequent MM-GBSA binding free energy calculations. The percentage of total output that was considered in the next step was as follows: from the initial 1000 compounds, 200 compounds were selected (with five poses for each) after the HTVS step, 50 compounds were selected (with five poses for each) after the SP step, and 10 compounds were selected (with five poses for each) after the XP step. These top 10 compounds with the best XP-Glide scores were then subjected for MM-GBSA assessment. Visualization of 2D protein–ligand interaction was done using Discovery Studio Visualizer V. 2022.

### MM-GBSA binding free energy calculation

Following the molecular docking analysis, the top ten indirubin derivatives with the highest XP-Glide scores, along with reference inhibitor OH8, were subjected to binding free energy calculations which incorporated the assessment based on top 3 binding poses using the Molecular Mechanics-Generalized Born Surface Area (MM-GBSA) method. MM-GBSA is an efficient and widely used approach for estimating binding free energies of protein–ligand complexes^26^. This step is crucial as it enables a more accurate estimation of the binding free energies of the ligands to the protein, taking into account the solvation effects and entropic contributions^27^. MM-GBSA calculations were performed using the Prime module in Schrödinger Maestro^18^. The complexes obtained from the XP docking results were utilized for these calculations. The binding free energy (∆GBind) of each complex was computed, including the contributions from molecular mechanics energies, solvation energies, and entropic effects. It combines molecular mechanics force fields with implicit solvent models to approximate the free energy of binding. The binding free energy (ΔGBind) of each protein–ligand complex was estimated using the OPLS3e force field^20^ and the VSGB 2.0 solvation model^28,29^. The obtained ∆GBind values were then used to rank the top hits based on their predicted binding affinities. By incorporating the MM-GBSA method, the study ensures a more reliable prediction of binding affinities compared to docking scores alone. This step allows for better differentiation between the top-ranked compounds and helps identify the most promising candidates for further evaluation through molecular dynamics simulations.

### Binding pose metadynamics simulation

To assess the stability of binding poses derived from molecular docking, we performed Binding Pose Metadynamics using Schrödinger Maestro^18^. The protein of interest was GSK3β (PDB ID: 6Y9R). The reference ligand for this study was OH8, and the top three indirubin derivatives were chosen based on their performance in docking and MM-GBSA assessments. For the metadynamics simulations, we selected the top three scoring poses as predicted by XP docking for each ligand. Each pose underwent 10 independent Metadynamics trials of 10 ns each to ensure robust statistical analysis^30^. The collective variable for these simulations was the root-mean-square deviation (RMSD) of the ligand from its initial pose, accounting for any drift by superimposing the binding sites across simulations. The Metadynamics panel in Desmond was utilized to evaluate the stability of the ligand-receptor complex. The stability was determined by monitoring the fluctuations of ligand RMSD and the persistence of key molecular interactions such as hydrogen bonds and pi interactions throughout the simulation duration. Multiple simulations were conducted to enhance statistical reliability, with the results averaged across these simulations to yield a comprehensive understanding of the binding pose stability. This plot is instrumental in visualizing the RMSD trends and determining the stability of the ligand within the active site of GSK3β during the simulations.

### E-pharmacophore feature mapping

E-pharmacophore feature mapping assessment was performed to identify the common structural and chemical features of the top three ranked indirubin derivatives obtained based on binding free energy difference (ΔGBind) energies from the previous step. For this, all the ligands were imported to Schrödinger Maestro^18^ and OH8, the reference GSK3β inhibitor used in this study, was selected to generate its pharmacophore features using the ‘Develop Pharmacophore model’ wizard in the Phase module^31,32^ of Schrödinger Maestro^18^. All possible features were included in developing the hypothesis. The pharmacophore hypothesis of OH8 and the top three indirubin derivatives was then generated in a similar manner. This set helped to identify the key pharmacophore features that were important for interacting with GSK3β. The pharmacophore feature superimposition assessment was then performed, where the pharmacophore features of OH8 and the top three indirubin derivatives while interacting with the GSK3β complex were extracted. These features of each ligand (OH8 and the top three ranked indirubin derivatives) interacting with GSK3β were superimposed to determine the similarity in their molecular ability to interact with GSK3β.

### Molecular dynamics (MD) simulations

MD simulations for 100 ns were performed to investigate the stability and dynamic behavior of the GSK3β-OH8 complex and the top three indirubin derivatives in complex with GSK3β using the Desmond module in Schrödinger Maestro^18^, with the OPLS3e force field^20^. MD simulations can provide insights into the conformational flexibility of the protein–ligand complexes, identify key interactions responsible for binding, and reveal potential allosteric effects or induced-fit phenomena^33^. Each protein–ligand complex was solvated in an orthorhombic box with TIP3P water molecules, and counter ions were added to neutralize the system. The systems were energy-minimized, heated, and equilibrated under NPT (constant number of particles, pressure, and temperature) conditions, maintaining a pressure of 1 atm and a temperature of 300 K using the Nose–Hoover thermostat^34^. Production MD simulations were run for 100 ns with a time step of 2 fs, and coordinates were saved every 10 ps for further analysis.

The ‘Simulation Interaction Diagram’ module in Desmond wizard of Schrödinger Maestro^18^ was used to post-process and analyze the trajectories of the MD simulations. The trajectories were aligned to the initial protein structure to eliminate the translational and rotational motions of the system. The RMSD of the protein backbone and the ligand heavy atoms as a function of time was calculated to evaluate the structural stability of each protein–ligand complex. The RMSD values were compared with the experimental B-factors to determine the agreement between the simulated and observed dynamics. The root-mean-square fluctuation (RMSF) of the protein residues as a function of time was calculated to assess the structural flexibility of each protein–ligand complex. The RMSF values were used to identify regions with higher or lower mobility and to relate them with the functional role of the protein^35^. Ligand–protein interactions, such as hydrogen bonds, hydrophobic contacts, and pi-stacking interactions, were analyzed to identify key residues involved in the binding of the ligands. The Protein–Ligand Interaction module was used to monitor the number and duration of these interactions throughout the simulation. The interaction profiles obtained from the MD simulations were compared with those from the initial docking calculations to evaluate the consistency and reliability of the predicted binding modes^36^.

### MM-GBSA assessment of MD simulation trajectories

MM-GBSA analysis was carried out post MD simulations. This assessment included top three hits of indirubin derivative, along with OH8, a reference inhibitor. MM-GBSA analysis of these compounds were examined in complex with GSK3β (PDB ID: 6Y9R). The MM-GBSA evaluations were conducted on the last 10 ns (ns) of the 100 ns MD simulations, specifically between 90 and 100 ns. These simulations were performed using Schrödinger’s Maestro^18^, utilizing the Desmond module as mentioned earlier. For the post-MD simulation MM-GBSA analysis, Schrödinger’s Python script ‘*thermal_mmgbsa.py*’ was employed. This script calculates the binding free energies of the ligand–protein complexes, an essential aspect of understanding the interaction dynamics. The general structure of the command used for running the script was “*run thermal_mmgbsa.py [input_file] -j energy-calc -start_frame [start_frame] -end_frame [end_frame] -step_size [step] -HOST localhost:[cores] -NJOBS [jobs]* > *[output_file]*”. In this study, the script was executed with a step size of 1 to analyze each frame from 90 to 100 ns, enhancing the resolution of the analysis. The resultant data encompassed various energy components, such as Binding energy (ΔGBind), Coulomb energy (ΔGCoulomb), Covalent bond energy (ΔGCovalent), Hydrogen-bonding correction (ΔGHbond), Lipophilic energy (ΔGLipo), Pi-Pi packing correction (ΔGPacking), Solvation energy (ΔGSolvation), and Van der Waals energy (ΔGvdW). To effectively visualize and interpret this data, violin plots were created. These plots were generated using Plotly Studio, a free and open-source online tool available at https://chart-studio.plotly.com/. The violin plots provided a comprehensive and graphical representation of the energy distribution and interactions within the studied ligand–protein complexes, offering valuable insights into their binding characteristics and energetics.

### In-silico pharmacokinetics

The ADMET (Absorption, Distribution, Metabolism, and Excretion–Toxicity) properties of the reference inhibitor of GSK3β (OH8) and the three top ranked indirubin derivatives were predicted using the pkCSM web server^37^. The pkCSM web server uses graph-based signatures to predict both physicochemical and pharmacological properties^37^. The SMILES strings of the compounds were obtained from PubChem^15–17^ and submitted to the pkCSM web server. The web server calculated the following in-vivo absorption parameters: water solubility in buffer system (SK atomic types, mg/L), Caco2 cell permeability (human colorectal carcinoma), human intestinal absorption (HIA, %), P-glycoprotein inhibition, and skin permeability (logKp, cm/hour). The web server also predicted the following in-vivo metabolic parameters: cytochrome P450 2C19 inhibition, cytochrome P450 2C9 inhibition, cytochrome P450 2D6 inhibition, cytochrome P450 2D6 substrate, cytochrome P450 3A4 inhibition, and cytochrome P450 3A4 substrate. The distribution property included tests for blood–brain barrier (BBB) penetration, Lipinski’s rule (rule of five), and central nervous system (CNS) permeability. The web server assessed the toxicity of the compounds under study by computing a range of important endpoints, such as acute algae toxicity, Ames test, 2 years carcinogenicity bioassay in mouse, 2 years carcinogenicity bioassay in rat, and in-vivo Ames test result in TA100 strain (metabolic activation by rat liver homogenate). Excretion is another crucial parameter as poor renal clearance often leads to the withdrawal of many drugs at clinical trial stages. In this study, total renal clearance and renal OCT2 substrate were included to identify the excretion efficacy of the proposed metabolite. Moreover, the web server provided chemical properties of each ligand such as Molecular Weight, LogP, #Rotatable Bonds, #Acceptors, #Donors, and Surface Area.

## Results

### Redocking of OH8 with GSK3β

**Figure 2:**
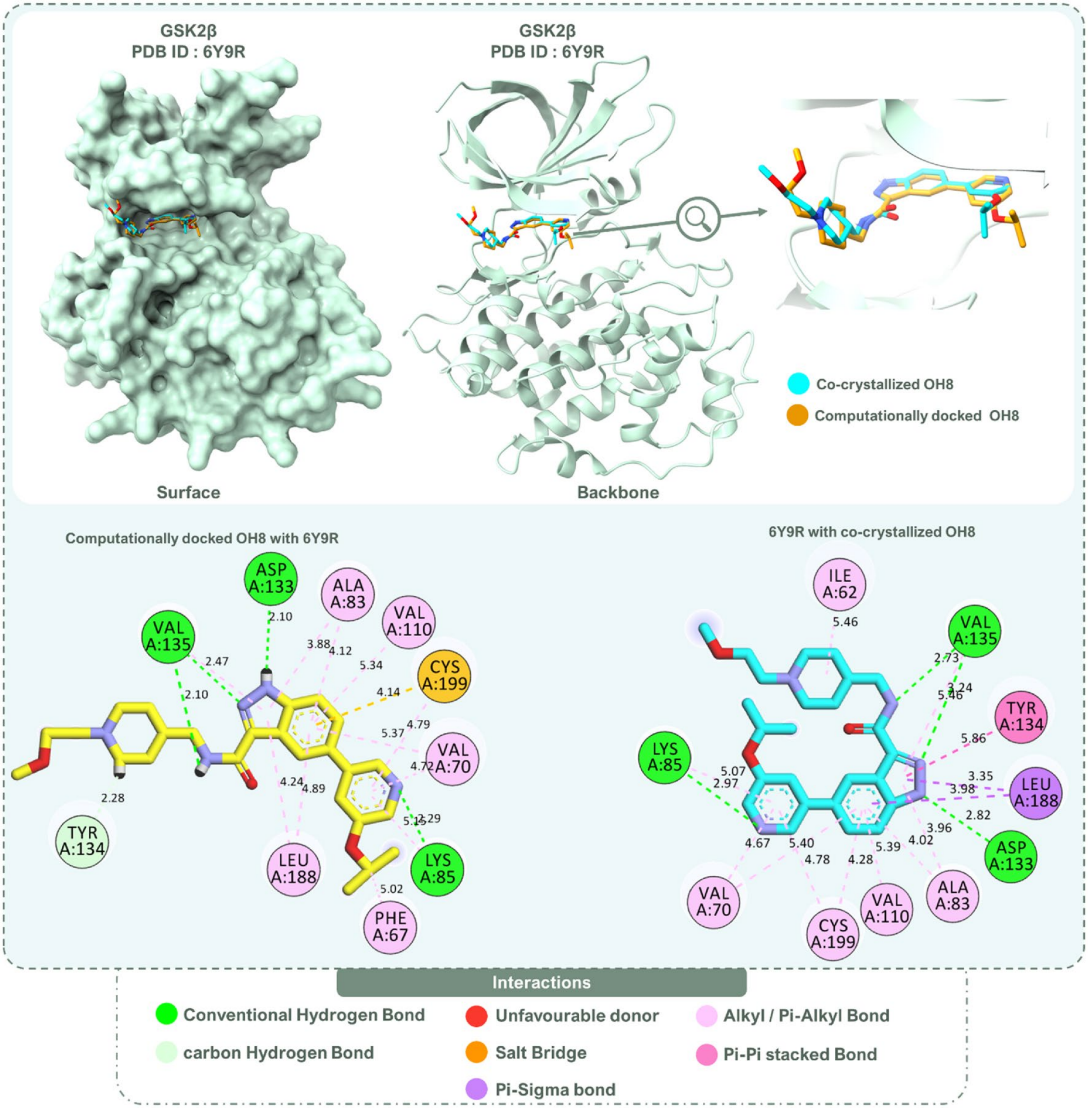
Redocking of reference inhibitor OH8 with GSK3β (PDB ID: 6Y9R) and comparison of best docked posed with co-crystalized OH8 in the retrieved protein. The values represented on the bond representation between amino-acids and ligand represents bond lengths in Angstron (Å).

To ascertain the accuracy and reliability of a docking method, redocking analysis is often employed. This analysis involves comparing the predicted poses of a known ligand with its experimental pose. Further, it serves to optimize both the docking parameters and scoring functions tailored for a particular receptor-ligand system. In this research, redocking was executed using Schrödinger Maestro V. 2021, and the resulting poses were visualized employing Discovery Studio Visualizer V. 2022 software. Identical results were observed upon juxtaposing the co-crystallized OH8 poses with the docked OH8 poses, as manifested by their superimposition in Fig. 2. Such a match is deemed commendable, signaling that the employed docking method has the prowess to emulate the experimental pose of OH8 with commendable accuracy and precision. Delving into the specifics of the protein, GSK3β (PDB ID: 6Y9R) was the chosen candidate for redocking, having been co-crystallized with the OH8. The protein data showcases a method derived from X-RAY DIFFRACTION, a fine resolution of 2.08 Å, an R-Value Free of 0.226, an R-Value Work of 0.189, and an R-Value Observed of 0.191. Significantly, these values are indicative of the protein’s superior quality, emphasizing its suitability for the study. Five poses emerged from the analysis, with the premier pose aligning most congruently with the co-crystallized ligand. This alignment was further validated with a docking XP GScore of -8.67 (kcal/mol), as recorded in Table 1. The minimal RMSD value of 0.36 Å between the redocked pose and the experimental binding mode of OH8 emphasizes the credibility of the docking protocol, deeming it adept for predicting the binding modes of indirubin derivatives. Figure 2 offers a detailed illustration, capturing the superimposition of the co-crystallized OH8, immersed in blue, against the backdrop of the redocked OH8, portrayed in yellow. Pivoting to the interaction dynamics, several contacts were observed between the co-crystallized ligand and its counterparts, with notable mentions being H-bonds with Lys85, Asp133, and Val135, among others. The docked OH8 mirrored these intricate interactions, with several of them being identical. Central to these interactions are the H-bonds with Asp133 and Val135, emphasized by the indazole moiety’s strategic anchoring at the adenine (of ATP) binding site of the protein. This engagement with hinge residues is illuminated through distinct hydrogen bonds, a spectacle captured vividly in Fig. 2, which also spotlights the co-crystallized and docked OH8 in close proximity. The insights gleaned from the redocking analysis instill a reinforced confidence in the method, paving the way for its application in subsequent docking explorations with the same system and methodology.

**Table 1:** Docking energies and MM-GBSA binding free energy change profiles of GSK3β with top ten indirubin derivatives.

Ranks	PubChem CID	Name	Docking XP score (Kcal/mol)	ΔGBind (Kcal/mol)	ΔGCoulomb (Kcal/mol)	ΔGCovalent (Kcal/mol)	ΔGHbond (Kcal/mol)	ΔGLipo (Kcal/mol)	ΔGPacking (Kcal/mol)	ΔGSolvation (Kcal/mol)
Reference inhibitor (OH8)	146036038	N-[[1-(2-methoxyethyl)piperidin-4-yl]methyl]-5-(5-propan-2-yloxypyridin-3-yl)-1H-indazole-3-carboxamide	− 8.67	− 56.54	30.23	9.59	− 1.96	− 22.65	− 0.87	− 14.15
1	137047232	1-[3-(benzotriazol-5-ylidenemethyl)-2-hydroxy-1H-indol-5-yl]-4-methylpentan-2-one	− 10.33	− 87.91	− 22.84	3.24	− 0.83	− 46.31	0.00	24.67
2	136618625	1-[2-hydroxy-3-[N-(4-methylpiperazin-1-yl)-C-phenylcarbonimidoyl]-1H-indol-5-yl]ethanone	− 10.74	− 80.93	9.83	1.62	− 0.93	− 37.78	0.00	− 4.88
3	136401024	3-[2-hydroxy-3-[(3-hydroxyphenyl)iminomethyl]-1H-indole-5-carbonyl]benzamide	− 11.97	− 78.86	− 37.17	9.17	− 2.48	− 29.99	− 0.67	26.88
4	136400954	4-[2-hydroxy-3-[(3-hydroxyphenyl)iminomethyl]-1H-indole-5-carbonyl]benzamide	− 12.42	− 77.82	− 31.85	4.33	− 1.85	− 29.83	− 0.89	28.19
5	136352004	5-(2-chloroacetyl)-3-quinolin-2(1H)-ylideneindolin-2-one	− 10.35	− 77.43	− 22.13	2.49	− 0.93	− 34.74	− 0.10	21.92
6	90901907	7-(5-fluoro-2-hydroxy-1H-indol-3-yl)-N-(2-hydroxyethyl)-2-methyl-4,5-dihydro-1H-indole-3-carboxamide	− 10.03	− 73.78	− 21.04	4.53	− 1.97	− 47.19	− 0.02	29.28
7	136502785	N-[3-[[(5-acetyl-2-hydroxy-1H-indol-3-yl)-phenylmethylidene]amino]propyl]ethanesulfonamide	− 10.59	− 70.23	24.96	7.38	− 0.75	− 38.64	− 0.56	− 16.03
8	136016428	2-hydroxy-3-(3-oxoindol-2-yl)-N-[(2S,3R,4R,5R)-2,3,4,5,6-pentahydroxyhexyl]-1H-indole-5-carboxamide	− 11.84	− 68.03	6.20	1.52	− 3.66	− 31.68	0.00	11.06
9	58125969	3-(3,4-dihydroxyphenyl)-1-(5-fluoro-1H-indol-2-yl)propan-1-one	− 10.13	− 64.97	− 28.76	1.57	− 1.27	− 26.66	− 0.18	24.39
10	11652264	(5-Fluoro-1H-indol-2-yl)-(5-hydroxy-1H-indol-2-yl)-methanone	− 10.15	− 62.33	− 23.68	0.22	− 0.57	− 25.55	− 0.19	21.38

ΔGBind = Binding energy, ΔGCoulomb = Coulomb energy, ΔGCovalent = Covalent bond energy, ΔGHbond = Hydrogen-bonding correction, ΔGLipo = Lipophilic energy, ΔGPacking = Pi-Pi packing correction, ΔGSolvation = Solvation energy.

### Filtering ligands by molecular docking by virtual screening workflow

Within the realm of computational biochemistry, the Virtual Screening Workflow stands as a pivotal technique, meticulously curated to identify potential therapeutic agents from vast molecular repositories. Its essence lies in binding affinity estimation and/or activity of these molecules against a designated target. For this endeavor, the workflow was harnessed to sift through an extensive array of a thousand indirubin derivatives, eventually refining this vast pool to a select ten. The GSK3β protein, vital to this study, served as the receptor, and the docking was executed at coordinates congruent with those of co-crystallized ligands.

Emerging at the forefront of this curation was the compound 4-[2-hydroxy-3-[(3-hydroxyphenyl)iminomethyl]-1H-indole-5-carbonyl]benzamide (PubChem CID: 136400954), with a stellar XP GScore of − 12.42. Its counterpart, 3-[2-hydroxy-3-[(3-hydroxyphenyl)iminomethyl]-1H-indole-5-carbonyl]benzamide (CID: 136401024), trailed closely, registering − 11.96, while the molecule 2-hydroxy-3-(3-oxoindol-2-yl)-N-[(2S,3R,4R,5R)-2,3,4,5,6-pentahydroxyhexyl]-1H-indole-5-carboxamide (CID: 136016428) unveiled an affinity of − 11.83. Yet, the narrative doesn't end here. Other noteworthy derivatives include 1-[2-hydroxy-3-[N-(4-methylpiperazin-1-yl)-C-phenylcarbonimidoyl]-1H-indol-5-yl]ethanone (CID: 136618625), N-[3-[[(5-acetyl-2-hydroxy-1H-indol-3-yl)-phenylmethylidene]amino]propyl]ethanesulfonamide (CID: 136502785), 5-(2-chloroacetyl)-3-quinolin-2(1H)-ylideneindolin-2-one (CID: 136352004), 1-[3-(benzotriazol-5-ylidenemethyl)-2-hydroxy-1H-indol-5-yl]-4-methylpentan-2-one (CID: 137047232), (5-Fluoro-1H-indol-2-yl)-(5-hydroxy-1H-indol-2-yl)-methanone (CID: 11652264), 3-(3,4-dihydroxyphenyl)-1-(5-fluoro-1H-indol-2-yl)propan-1-one (CID: 58125969), and 7-(5-fluoro-2-hydroxy-1H-indol-3-yl)-N-(2-hydroxyethyl)-2-methyl-4,5-dihydro-1H-indole-3-carboxamide (CID: 90901907) (Table 1), each echoing its unique therapeutic potential.

These XP GScores, while instrumental, are merely the beginning. To truly discern the therapeutic potential of these compounds, a multi-faceted analysis is essential. Consequently, these derivatives will undergo the rigorous MM-GBSA paradigm in subsequent phases, offering a more nuanced and comprehensive re-scoring and ranking, ensuring their holistic evaluation as potential therapeutic agents.

### Evaluation of indirubin derivatives for GSK3β inhibition through MM-GBSA

In the intricate arena of bioinformatics, this study embarked on a mission to dissect the binding dynamics of indirubin derivatives with GSK3β. Utilizing both docking and MM-GBSA methodologies, the binding affinity and free energy profiles of these derivatives were scrutinized, with OH8 serving as a pivotal reference compound. The data spotlighted the delicate balance between the docking XP GScore, which reflects the binding affinity, and the MM-GBSA ΔGBind, which delves into the binding free energy alterations upon complex formation. This ΔGBind is a composite of myriad components such as ΔGCoulomb, ΔGCovalent, ΔGHbond, ΔGLipo, ΔGPacking, ΔGSolvation, and ΔGvdW, each shedding light on distinct interaction dynamics between GSK2β and the ligands.

Delving deeper into the data, the narrative showcases a compelling interplay of these scores, unraveling the molecular intricacies of the GSK3β and indirubin derivative interactions. The reference compound, OH8, charted an XP GScore of − 8.67 and an MM-GBSA score of − 56.54 kcal/mol. However, the derivatives painted a more nuanced picture. The compound 1-[3-(benzotriazol-5-ylidenemethyl)-2-hydroxy-1H-indol-5-yl]-4-methylpentan-2-one (Rank 1, PubChem CID: 137047232) ascended to the forefront with an XP GScore of − 10.33 and an MM-GBSA score of − 87.91 kcal/mol. It was closely flanked by 1-[2-hydroxy-3-[N-(4-methylpiperazin-1-yl)-C-phenylcarbonimidoyl]-1H-indol-5-yl]ethanone (Rank 2, CID: 136618625) and 3-[2-hydroxy-3-[(3-hydroxyphenyl)iminomethyl]-1H-indole-5-carbonyl]benzamide (Rank 3, CID: 136401024), underscoring their potent profiles (Table 1).

It is paramount to recognize that while a compound might register a relatively lower docking score, its MM-GBSA score could be more commendable. This apparent dichotomy arises due to the differential paradigms these techniques operate on. Docking primarily captures the geometric congruence between the ligand and receptor, occasionally glossing over intricate energetic facets. On the other hand, MM-GBSA offers a more refined perspective, by including solvation dynamics, entropy considerations, and detailed energy interplays, thus it provides a more robust and reliable metric than mere docking scores.

Given their exemplary profiles, the top three derivatives have been earmarked for rigorous scrutiny. To further validate their therapeutic prospects, these elite contenders will undergo 100 ns MD simulations, aiming to chronicle their temporal interactions with GSK3β and potentially reaffirming their position as potent GSK3β inhibitors.

### Deep dive into the protein–ligand interactions of indirubin derivatives with GSK2β

In the complex realm of molecular interactions, it is essential to thoroughly comprehend the detailed mechanisms of protein–ligand interactions to fully appreciate and leverage the therapeutic potential of molecular entities. This study, in its endeavor, meticulously analyzed the interaction profiles of select indirubin derivatives with GSK2β, drawing parallels with the established reference inhibitor, OH8.

OH8, as the reference, established itself as a gold standard by showcasing a diverse spectrum of interactions with GSK2β. The salient features of its binding profile encompass hydrogen bonds with key residues like Lys85, Asp133, and Val135. Augmenting this, a complex network of Alkyl/Pi-Alkyl interactions and a prominent salt bridge with Cys199 emerged. Furthermore, Carbon hydrogen bonds with Tyr134 provided added depth to its interaction matrix, as illustrated in Fig. 2.

**Figure 3:**
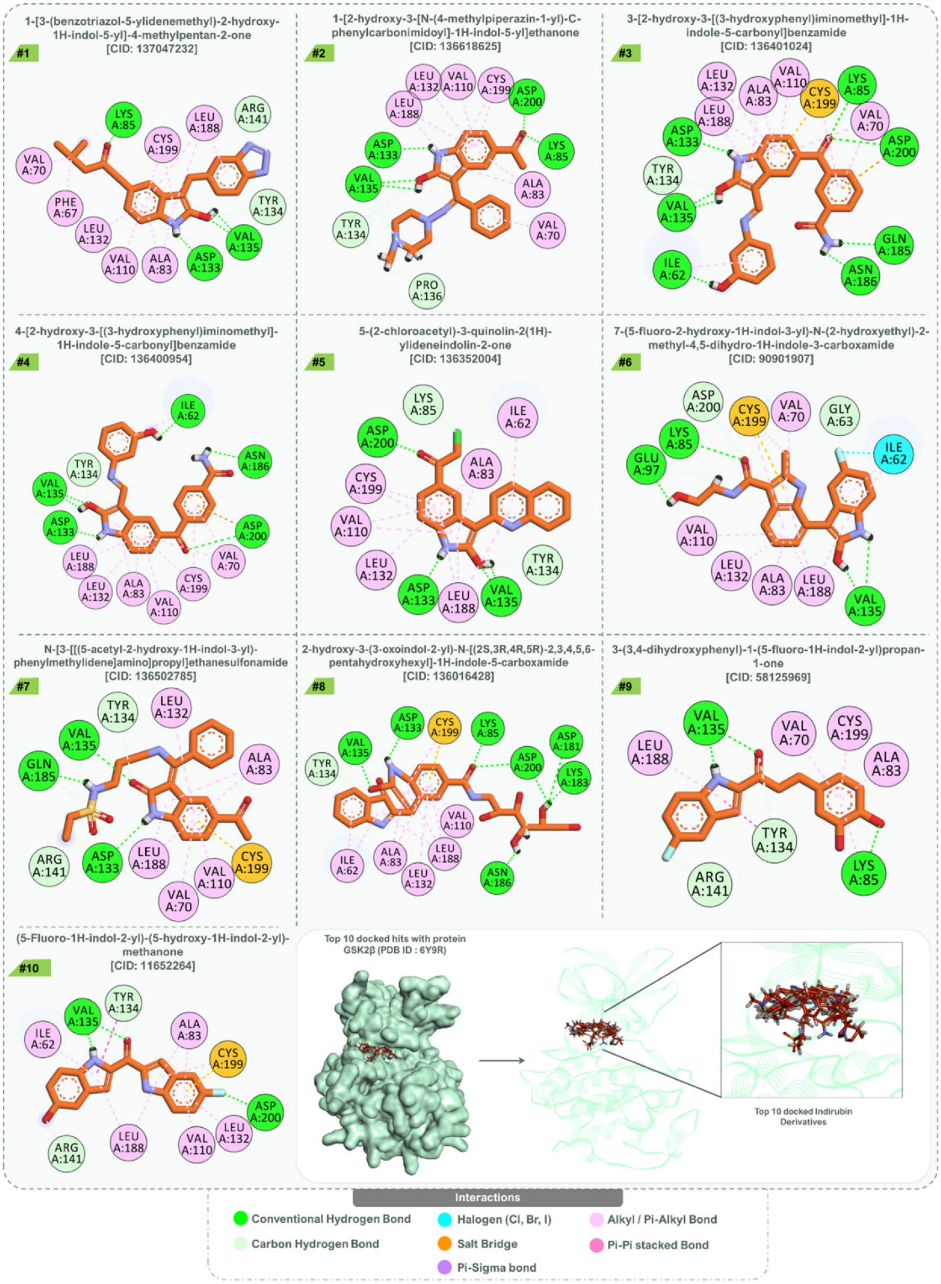
Interaction pattern of docked top 10 indirubin derivatives with GSK3β (PDB ID: 6Y9R).

Reflecting a similar interaction paradigm, the compound 1-[3-(benzotriazol-5-ylidenemethyl)-2-hydroxy-1H-indol-5-yl]-4-methylpentan-2-one (Rank 1) drew parallels with OH8, especially with its hydrogen bonds involving Lys85, Val135, and Asp133. A tapestry of Alkyl/Pi-Alkyl interactions with residues like Phe67, Val70, and Ala83 mirrored the dynamics seen in OH8. Of note, Carbon hydrogen bonds with Tyr134 and Arg141 enriched its interaction landscape. Following closely, the derivative 1-[2-hydroxy-3-[N-(4-methylpiperazin-1-yl)-C-phenylcarbonimidoyl]-1H-indol-5-yl]ethanone (Rank 2) echoed the quintessential hydrogen bonding with Lys85, Val135, and Asp133, while also reaching out to Asp200. Its Alkyl/Pi-Alkyl interactions, evoking memories of OH8’s profile, along with Carbon hydrogen bonds with Tyr134 and Pro136, added layers of complexity. The compound 3-[2-hydroxy-3-[(3-hydroxyphenyl)iminomethyl]-1H-indole-5-carbonyl]benzamide (Rank 3) further expanded the interaction horizon, especially with its hydrogen bonds to Ile62, Lys85, and Asp133, among others. Its binding dynamics, particularly the Carbon hydrogen bond with Tyr134, harmonized well with OH8. The interaction intricacies of these compounds are vividly depicted in Fig. 3. While interactions of compounds ranked 4 to 10 are also captured in Fig. 3, the narrative primarily emphasizes the top three ranks, given their selection for further pharmacophore feature mapping and MD-simulations in juxtaposition with OH8. To encapsulate, the symphonic interaction patterns between the indirubin derivatives and OH8 underscore their potential therapeutic avenues. Their finesse in mirroring and at times transcending the interaction prowess of OH8 with GSK2β positions them as compelling candidates in therapeutic research.

### Binding pose metadynamics simulation

**Figure 4:**
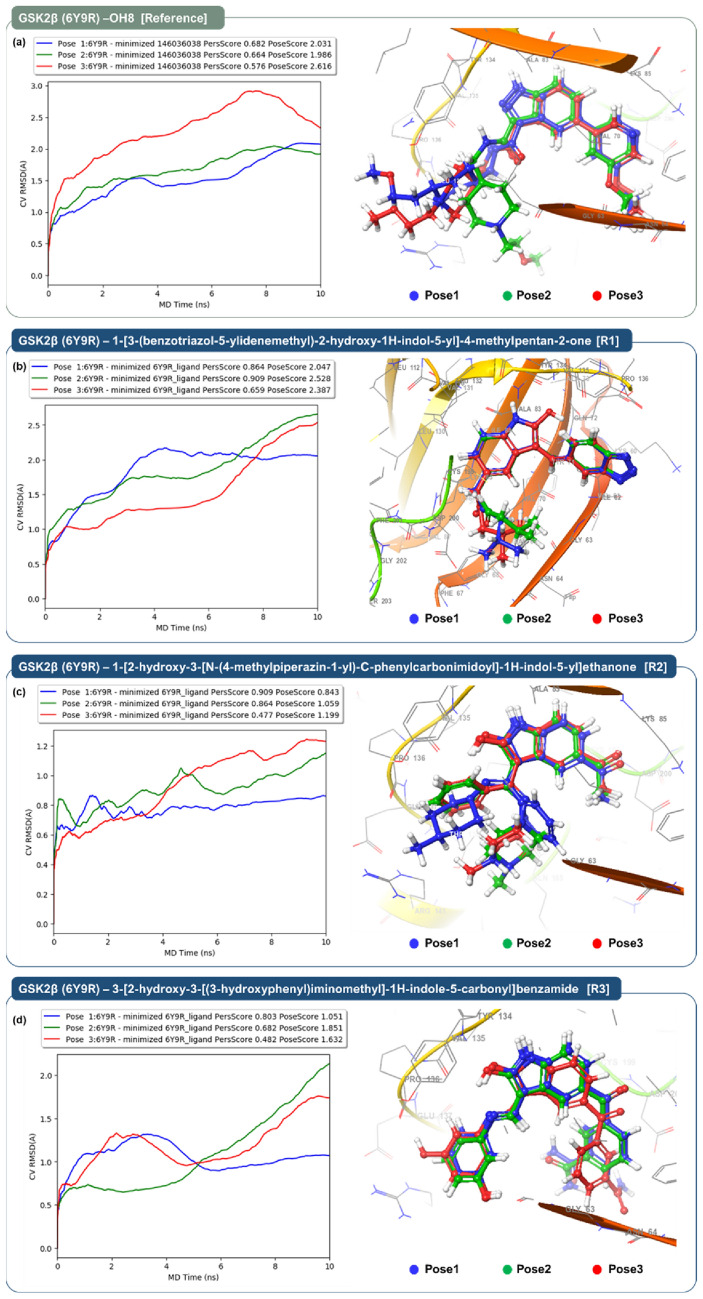
Plots of RMSD from binding pose metadynamics assessments of OH8, 1-[3-(benzotriazol-5-ylidenemethyl)-2-hydroxy-1H-indol-5-yl]-4-methylpentan-2-one (Rank 1), 1-[2-hydroxy-3-[N-(4-methylpiperazin-1-yl)-C-phenylcarbonimidoyl]-1H-indol-5-yl]ethanone (Rank 2) and 3-[2-hydroxy-3-[(3-hydroxyphenyl)iminomethyl]-1H-indole-5-carbonyl]benzamide (Rank 3) in complex with GSK3β (PDB ID: 6Y9R).

The outcomes of the Binding Pose Metadynamics for the top three poses of the reference compound OH8 and the top three ranked indirubin derivatives, complexed with GSK3β, are illustrated in Fig. 4. In Fig. 4a, Binding Pose Metadynamics for the reference compound OH8 complexed with GSK3β (PDB ID: 6Y9R) reveals Pose 1 (blue line) as the most stable with an RMSD steady around 1.5 Å, a PersScore of 0.682, and a PoseScore of 2.031, indicating robust retention of its docked conformation. Pose 2 (green line) displays moderate RMSD oscillations between 1.0 and 2.0 Å, with PersScore and PoseScore values of 0.664 and 1.986, respectively, suggesting a balance of stability and adaptability. Conversely, Pose 3 (red line) shows increased RMSD variability, ascending beyond 2.5 Å, which, despite its favourable scores (PersScore 0.576 and PoseScore 2.616), points to a less consistent binding interaction. The metadynamics thus substantiate Pose 1, identified by XP docking, as the most promising binding mode.

Figure 4b displays the Binding Pose Metadynamics results for the GSK3β complex with the Rank 1 ligand, revealing Pose 1 (blue line) as the most stable, with an RMSD value stabilizing around 2.0 Å, complemented by a PersScore of 0.864 and a PoseScore of 2.047, indicative of sustained binding conformation. Pose 2 (green line) demonstrates moderate RMSD fluctuations with scores of 0.909 for PersScore and 2.528 for PoseScore, suggesting adaptable interactions within the binding site. In contrast, Pose 3 (red line) shows increased RMSD movement and higher scores (PersScore of 0.659 and PoseScore of 2.387), pointing to a potentially less stable interaction. Collectively, these findings corroborate the XP docking results, with Pose 1 distinguished as the most viable conformation for stable ligand binding in GSK3β.

Figure 4c demonstrates the Binding Pose Metadynamics for the Rank 2 compound in complex with GSK3β, assessed through the RMSD values. Pose 1 (blue line) exhibits remarkable stability with an RMSD under 0.6 Å, along with a PersScore of 0.909 and a PoseScore of 0.843, suggesting a highly stable interaction with the protein. Pose 2 (green line) maintains moderate stability, with RMSD values fluctuating around 0.8 Å and scoring metrics of 0.864 for PersScore and 1.059 for PoseScore, indicating a reliable yet slightly more flexible binding mode. Pose 3 (red line), with an RMSD that progressively increases to just over 1.0 Å and scores of 0.477 for PersScore and 1.199 for PoseScore, implies a less stable interaction relative to the other poses. These metadynamics results align with the XP docking predictions, affirming that Pose 1 of the Rank 2 compound offers the most stable binding conformation within the GSK3β active site.

Figure 4d illustrates the Binding Pose Metadynamics for the Rank 3 compound in complex with GSK3β, showcasing the RMSD behavior. Pose 1 (blue line) displays an initial increase in RMSD but stabilizes around 1.0 Å, reflecting a stable interaction with the enzyme, as supported by a PersScore of 0.803 and a PoseScore of 1.051. Pose 2 (red line) shows greater variability, with RMSD rising to approximately 1.5 Å, indicative of less conformational stability despite a reasonable PersScore of 0.682 and PoseScore of 1.851. Finally, Pose 3 (green line) reveals a continuous increase in RMSD, suggesting significant conformational flexibility, which is further evidenced by the lowest PersScore of 0.482 and a PoseScore of 1.632. The data collectively suggest that Pose 1 maintains the most stable and potentially relevant conformation for the Rank 3 ligand within the GSK3β binding site.

### Comparative pharmacophore analysis of OH8 and top-ranked indirubin derivatives

Pharmacophore modelling is an indispensable tool in the realm of drug discovery, offering a window into the complexities of molecular features that play pivotal roles in ligand-receptor interactions. Against this backdrop, the study embarked on a detailed examination of the pharmacophore features of OH8, the reference molecule, in juxtaposition with three paramount indirubin derivatives. This endeavour aimed to glean insights into their potential binding dynamics with GSK3β (PDB ID: 6Y9R).

**Figure 5:**
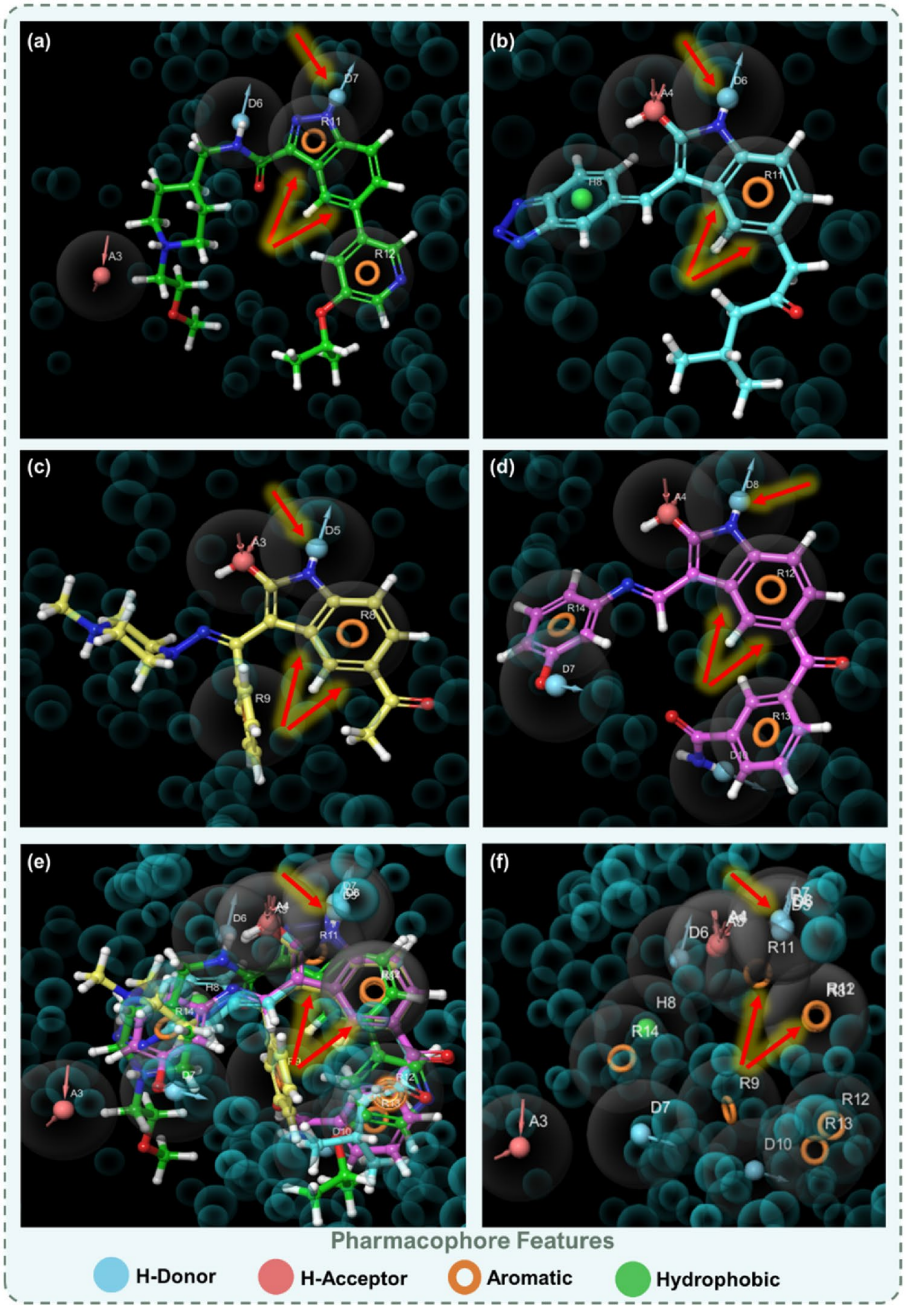
Pharmacophore Features of (**a**) OH8, (**b**) 1-[3-(benzotriazol-5-ylidenemethyl)-2-hydroxy-1H-indol-5-yl]-4-methylpentan-2-one (Rank 1), (**c**) 1-[2-hydroxy-3-[N-(4-methylpiperazin-1-yl)-C-phenylcarbonimidoyl]-1H-indol-5-yl]ethanone (Rank 2) and (**d**) 3-[2-hydroxy-3-[(3-hydroxyphenyl)iminomethyl]-1H-indole-5-carbonyl]benzamide (Rank 3) while interacting with GSK3β (PDB ID: 6Y9R), and arrows in Red indicate identical features amongst all the ligands, including reference inhibitor and top ranked indirubin derivatives, (**e**) Superposed image of all the ligands and their features and (**f**) Superposed image of only pharmacophore features of all the ligands.

Figure 5 provides a comprehensive visual representation of this detailed analysis. Panel (a) delineates the pharmacophore framework of OH8, serving as the cornerstone for subsequent comparative assessments. Progressing sequentially, panels (b) through (d) spotlight the pharmacophoric attributes of the indirubin derivatives. Specifically, panel (b) captures the features of 1-[3-(benzotriazol-5-ylidenemethyl)-2-hydroxy-1H-indol-5-yl]-4-methylpentan-2-one (Rank 1), panel (c) sheds light on 1-[2-hydroxy-3-[N-(4-methylpiperazin-1-yl)-C-phenylcarbonimidoyl]-1H-indol-5-yl]ethanone (Rank 2), and panel (d) illuminates the characteristics of 3-[2-hydroxy-3-[(3-hydroxyphenyl)iminomethyl]-1H-indole-5-carbonyl]benzamide (Rank 3).

Across all representations, two features were consistently observed: a hydrogen donor feature, represented by a blue sphere, and an aromatic moiety indicated by a dual-ring structure. The presence of these elements across the reference molecule OH8 and the leading indirubin derivatives suggests similar binding interactions with GSK3β, hinting at their potential efficacy. The visual overlay presented in Fig. 5 panel (e) offers a comparative analysis of these ligands, highlighting both shared characteristics and subtle variances, thereby providing a comprehensive view of their binding mechanisms. The subsequent panel (f) further isolates these pharmacophore features, abstracting them from the ligand backbones to focus on their interaction capabilities. Their striking similarities of top hits of indirubin derivatives with the reference molecule, OH8, suggest that they might not only emulate but also potentially enhance the therapeutic efficacies demonstrated by OH8. This approach seeks to delineate the molecular underpinnings that could render these derivatives as promising therapeutic agents.

### MD simulations

Following the XP docking procedure, the top 10 derivatives were further subjected to MM-GBSA assessments, subsequently leading to their ranking from 1 to 10, as depicted in Table 1. The first three ranks were secured by 1-[3-(benzotriazol-5-ylidenemethyl)-2-hydroxy-1H-indol-5-yl]-4-methylpentan-2-one (Rank 1), 1-[2-hydroxy-3-[N-(4-methylpiperazin-1-yl)-C-phenylcarbonimidoyl]-1H-indol-5-yl]ethanone (Rank 2), and 3-[2-hydroxy-3-[(3-hydroxyphenyl)iminomethyl]-1H-indole-5-carbonyl]benzamide (Rank 3). Remarkably, these compounds interacted with all pivotal amino acids that were engaged by the reference drug, OH8.

**Figure 6:**
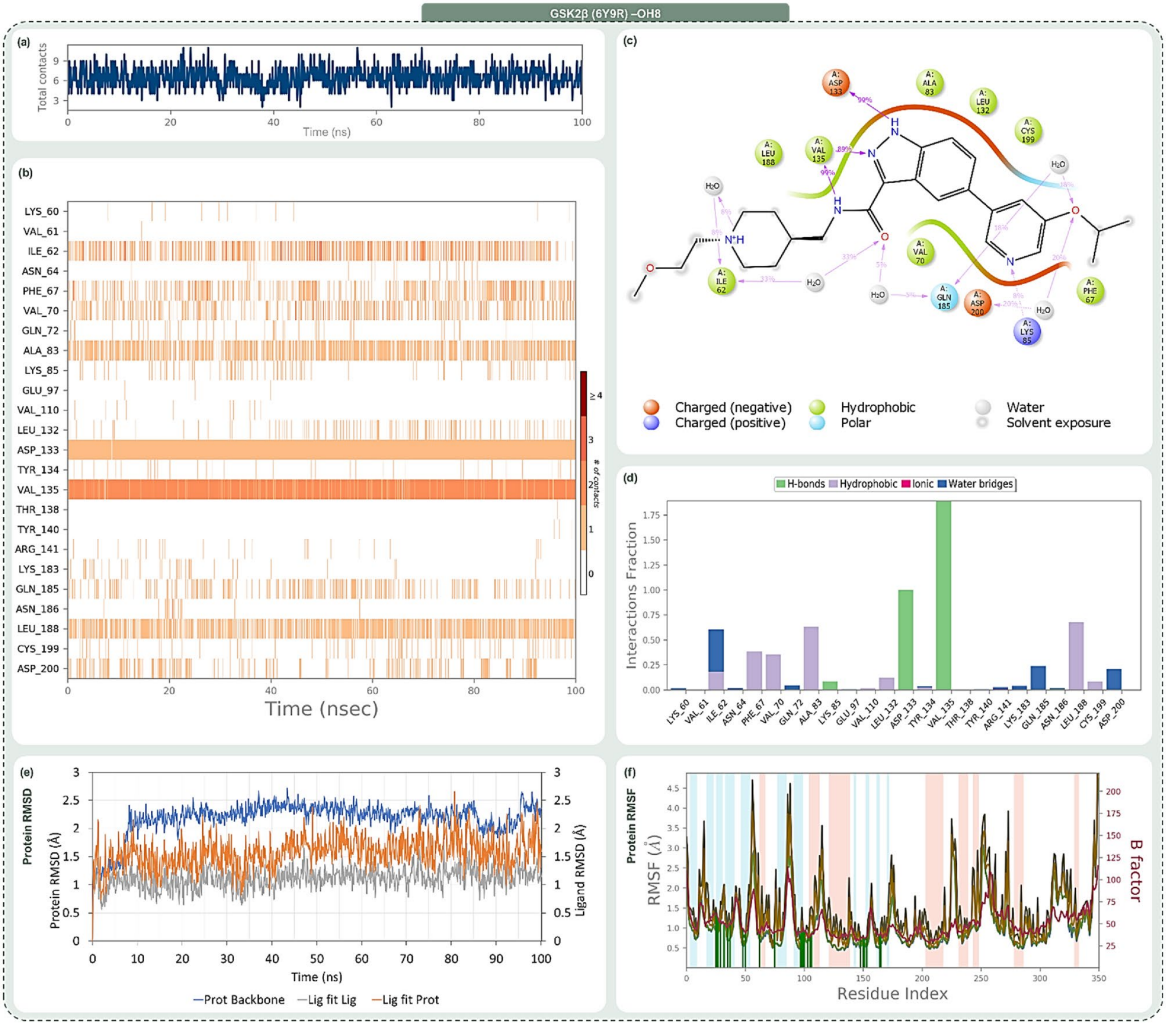
Post MD simulation assessment showing (**a**) total contacts, (**b**) amino acid interaction timeline, (**c**) percent interaction profile and (**d**) interaction fraction profile, (**e**) Protein–Ligand RMSD and (**f**) Protein RMSF of GSK3β (PDB ID: 6Y9R)—OH8 (Reference inhibitor) complex.

Delving deeper into their interaction profiles, Rank 1 mirrored OH8 by forming hydrogen bonds with Lys85, Val135, and Asp133. Rank 2, in addition to these conserved interactions, extended its bond formation to Asp200. Rank 3 reinforced its alignment with OH8 by securing hydrogen bonds with amino acids like Lys85, Asp133, and Val135. The congruence in interaction dynamics between these top compounds and OH8 not only underscores their potential therapeutic relevance but also posits them as promising analogous to OH8 in terms of biological activity. Given the compelling evidence from the docking scores, MM-GBSA evaluations, and interaction fidelity with GSK2β, the top three ranked compounds were selected for molecular dynamics (MD) simulations. The post-simulation evaluations of GSK2β-OH8 and GSK2β-indirubin derivative complexes, as illustrated in Figs. 6 through Fig. 9, are divided into four insightful panels per ligand: total contacts, amino acid interaction timeline, percent interaction profile, and interaction fraction profile. These visual representations decode the temporal progression of protein–ligand interactions and their binding robustness. The total contacts and amino acid interaction timeline delineate the concurrent contacts between the ligand and protein, with intensified orange hues signifying multi-point interactions. The percent interaction profile spotlights ligand atom engagements with protein residues that persist for over 10% of the simulation’s duration. Lastly, the interaction fraction profile dissects the nature of protein–ligand engagements into categories like Hydrogen Bonds, Hydrophobic interactions, Ionic bonds, and Water Bridges, visualized as stacked bar charts. These charts quantify the interaction persistence, with values surpassing 1.0 indicating instances where a single protein residue forms multiple interactions of a similar kind with the ligand.

**Figure 7:**
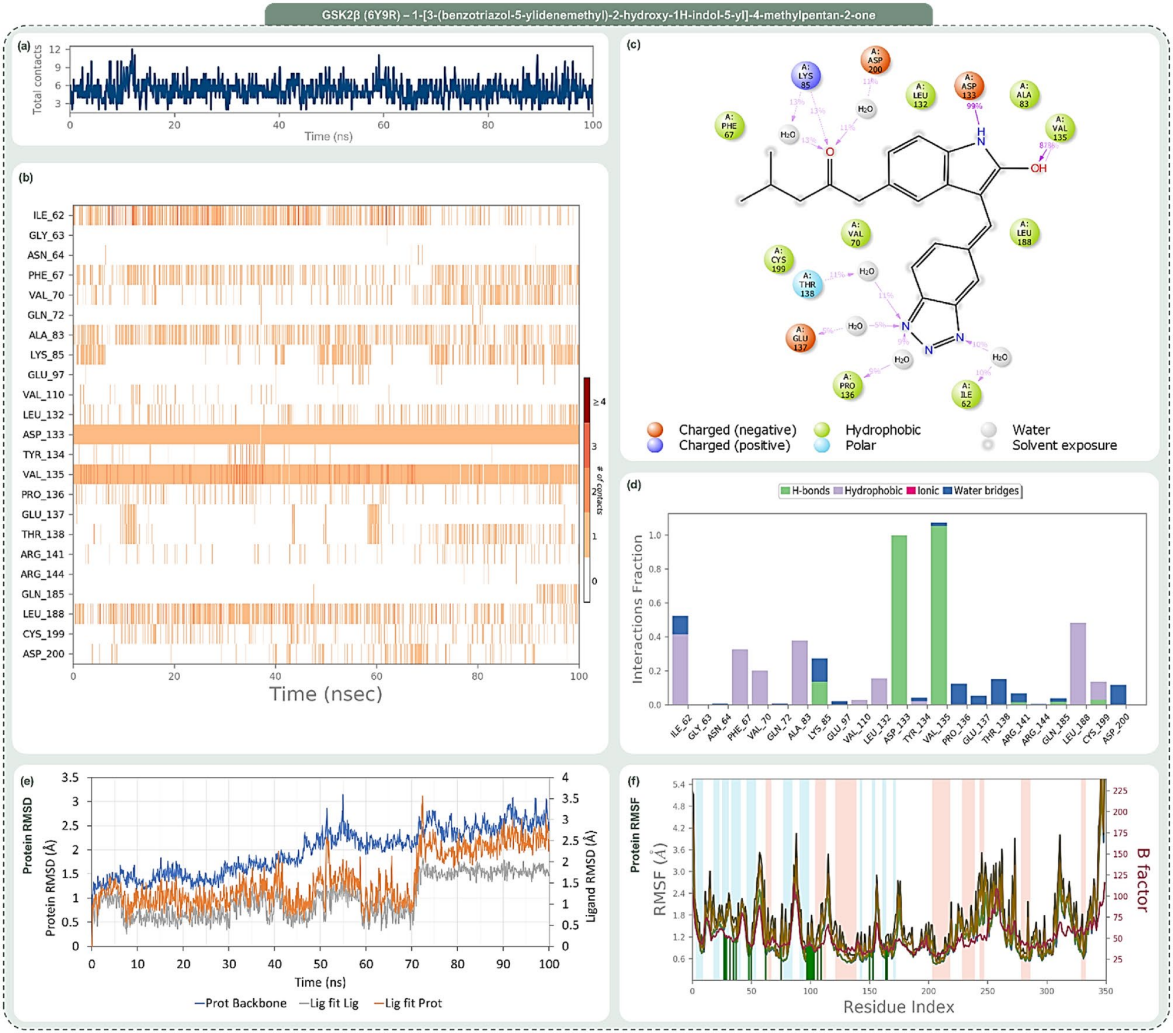
Post MD simulation assessment showing (**a**) total contacts, (**b**) amino acid interaction timeline, (**c**) percent interaction profile and (**d**) interaction fraction profile, (**e**) Protein–Ligand RMSD and (**f**) Protein RMSF of GSK3β (PDB ID: 6Y9R)—1-[3-(benzotriazol-5-ylidenemethyl)-2-hydroxy-1H-indol-5-yl]-4-methylpentan-2-one (Rank 1) complex.

**Figure 8:**
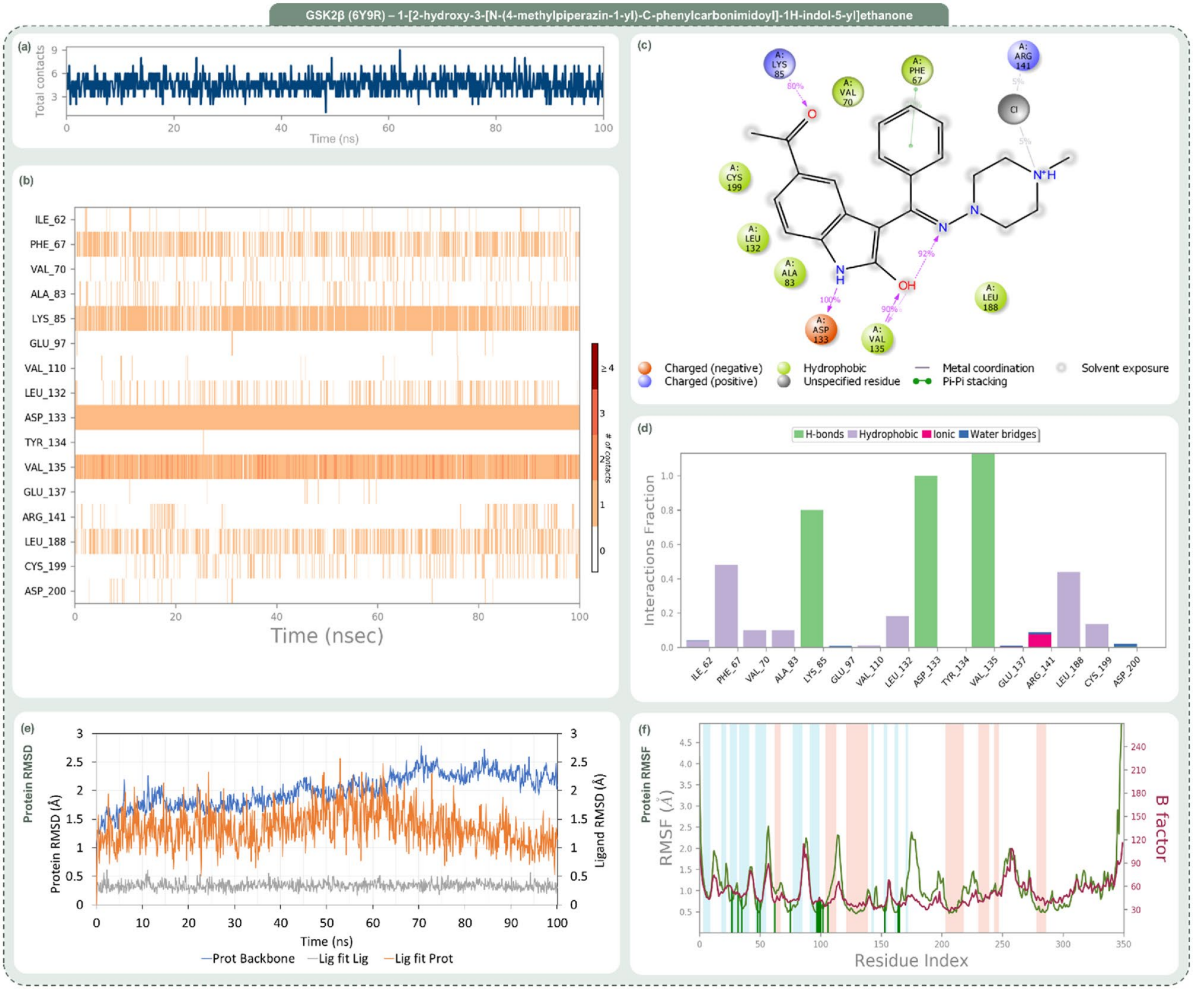
Post MD simulation assessment showing (**a**) total contacts, (**b**) amino acid interaction timeline, (**c**) percent interaction profile and (**d**) interaction fraction profile, (**e**) Protein–Ligand RMSD and (**f**) Protein RMSF of GSK3β (PDB ID: 6Y9R)—1-[2-hydroxy-3-[N-(4-methylpiperazin-1-yl)-C-phenylcarbonimidoyl]-1H-indol-5-yl]ethanone (Rank 2) complex.

**Figure 9:**
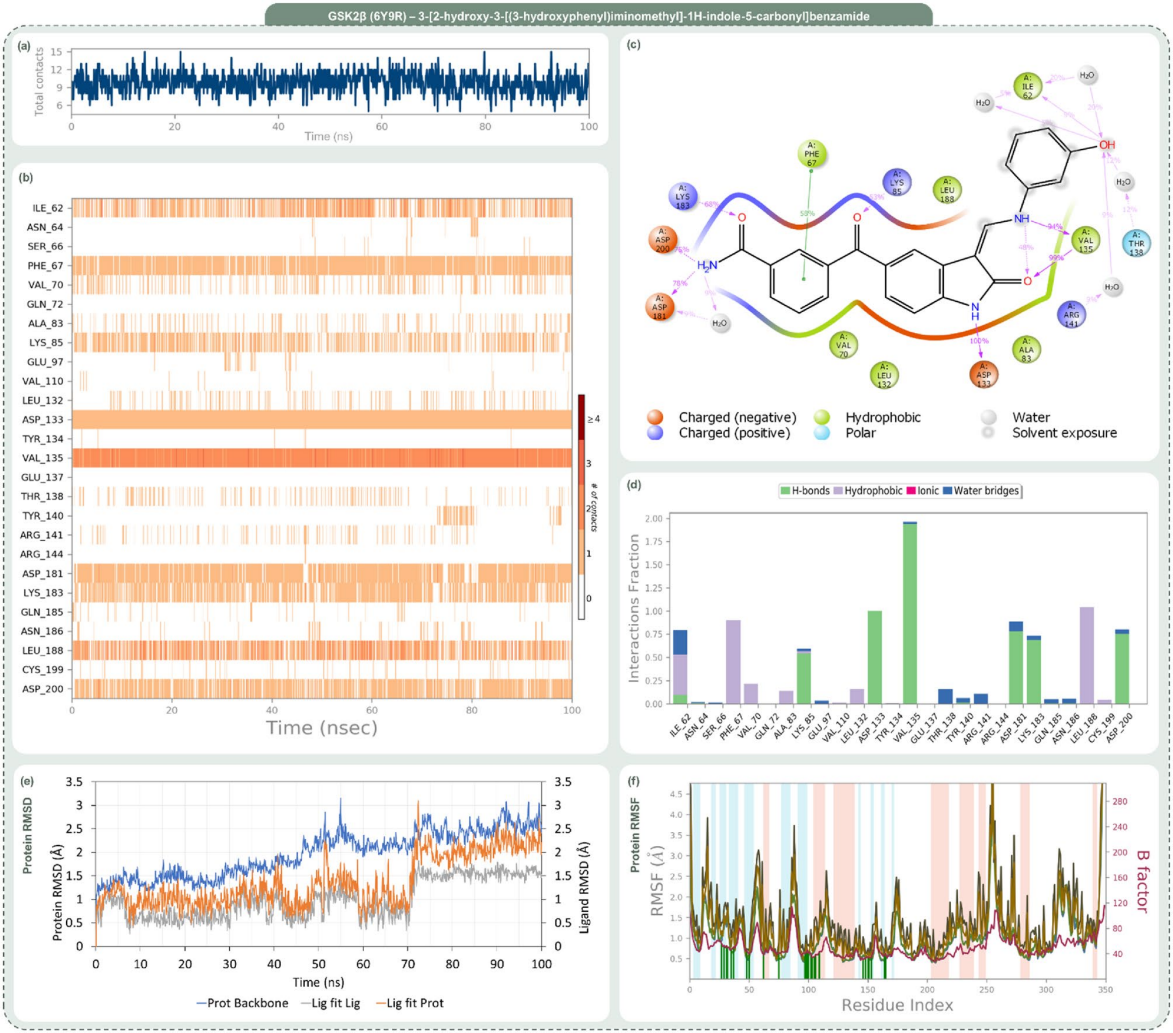
Post MD simulation assessment showing (**a**) total contacts, (**b**) amino acid interaction timeline, (**c**) percent interaction profile and (**d**) interaction fraction profile, (**e**) Protein–Ligand RMSD and (**f**) Protein RMSF of GSK3β (PDB ID: 6Y9R)—3-[2-hydroxy-3-[(3-hydroxyphenyl)iminomethyl]-1H-indole-5-carbonyl]benzamide (Rank 3) complex.

Figures 6, 7, 8 and 9 systematically represent the complex interactions between GSK2β and its ligand partners, capturing their dynamic behaviour throughout a 100 ns molecular dynamics (MD) simulation. Figure 6 sheds light on the nuanced engagement of the GSK2β-OH8 complex. The total contacts, illustrated in Fig. 6a, fluctuate between 2 and 10, with an average of 6, underscoring the fluid nature of protein–ligand interactions. The amino acid interaction timeline in Fig. 6b emphasizes persistent interactions with residues such as Asp133 and Val135. OH8’s diverse interaction spectrum, evident through its engagement with residues like Ile62, Phe67, and Gln185 among others, is highlighted. The intensity, denoted by the depth of the orange hue, underscores multiple touchpoints some residues have with the ligand. The percent interaction profile in Fig. 6c spotlights Asp133’s almost constant engagement (99%) and Val135’s multifaceted interaction, culminating in a cumulative percentage of 188%. Figure 6d classifies these interactions into types, revealing significant interaction fractions with residues like Ile62 and Val135, occasionally exceeding a value of 1.0 due to multifaceted interactions of the same type.

In Fig. 7, the GSK2β-Rank 1 complex interaction is in focus. Molecular dynamics shows a dynamic interaction pattern, as depicted in Fig. 7a, with contact points ranging from 2 to 11 and an average of 6. Asp133 and Val135 continue their steadfast engagement, with the Rank 1 compound widening its interaction purview to include residues like Ile62 and Pro136 as depicted in Fig. 7b. Figure 7c highlights Asp133’s consistent 99% interaction, with Val135’s interactions aggregating to 110%. The interaction fraction profile, Fig. 7d, magnifies interactions with residues like Ile62 and Lys85. The striking similarity of Rank 1 to the reference OH8 is underscored by these interactions.

Figure 8, which illustrates the dynamic interactions within the GSK2β-Rank 2 complex, reveals a range of 2 to 9 total contact points as demonstrated in Fig. 8a. Beyond the consistent interactions with Asp133 and Val135, Rank 2 garners strong interactions with residues like Phe67 and Lys85, as detailed in Fig. 8b. The percent profile in Fig. 8c highlights the heightened interaction with Lys85 (80%). The interaction fraction profile in Fig. 8d brings forth dominant interactions with residues like Phe67 and Lys85, emphasizing Rank 2’s resemblance to OH8, especially with added engagements.

Finally, Fig. 9 provides a detailed analysis of the interaction dynamics between the GSK2β-Rank 3 complex. The dynamism is palpable with total contacts, as shown in Fig. 9a, oscillating between 3 and 15, averaging at 9. The amino acid timeline in Fig. 9b accentuates the consistent bonds with Asp133 and Val135, while introducing enhanced interactions with residues like Asp181. The interaction percentages, depicted in Fig. 9c, spotlight Asp133’s unwavering 100% engagement and Val135’s amplified 193% interaction. The interaction fraction profile in Fig. 9d emphasizes significant engagements with residues such as Ile62 and Asp133. Rank 3 not only mirrors OH8’s interaction dynamics but also introduces additional robust interactions, enhancing its interaction portfolio.

The trajectory analysis of the 100 ns molecular dynamics (MD) simulation, focusing on RMSD and RMSF of GSK2β-ligand complexes encompassing OH8, Rank 1, Rank 2, and Rank 3, is delineated in Panel (e) and (f) of Figs. 6 through 9. The RMSD panel chronicles the mean displacement changes of selected atoms per frame relative to a reference, offering a window into the protein’s evolving structural conformation during the simulation. Typically, RMSD values that fluctuate between 1 and 3 Å, relative to the reference frame, are considered indicative of stability in compact, globular proteins, with deviations larger than this range suggesting significant conformational changes. The ligand RMSD, tagged as ‘Lig fit Prot’, portrays the ligand’s stability vis-à-vis the protein and its docking site. Discrepancies vastly greater than the protein RMSD potentially indicate the ligand’s drift from its proposed binding mode. The RMSF panel paints a picture of localized transformations along the protein sequence, with prominent peaks signifying the most variable segments during the simulation. Features like alpha-helices and beta-strands are accentuated, while ligand-interacting protein residues are demarcated with verdant vertical stripes. The protein’s RMSF can be juxtaposed with the experimental x-ray B-factor, although an exact match isn't anticipated. Summarily, the RMSD and RMSF metrics for all complexes remain within acceptable bounds, underscoring their stability across the 100 ns MD simulation span.

**Figure 10:**
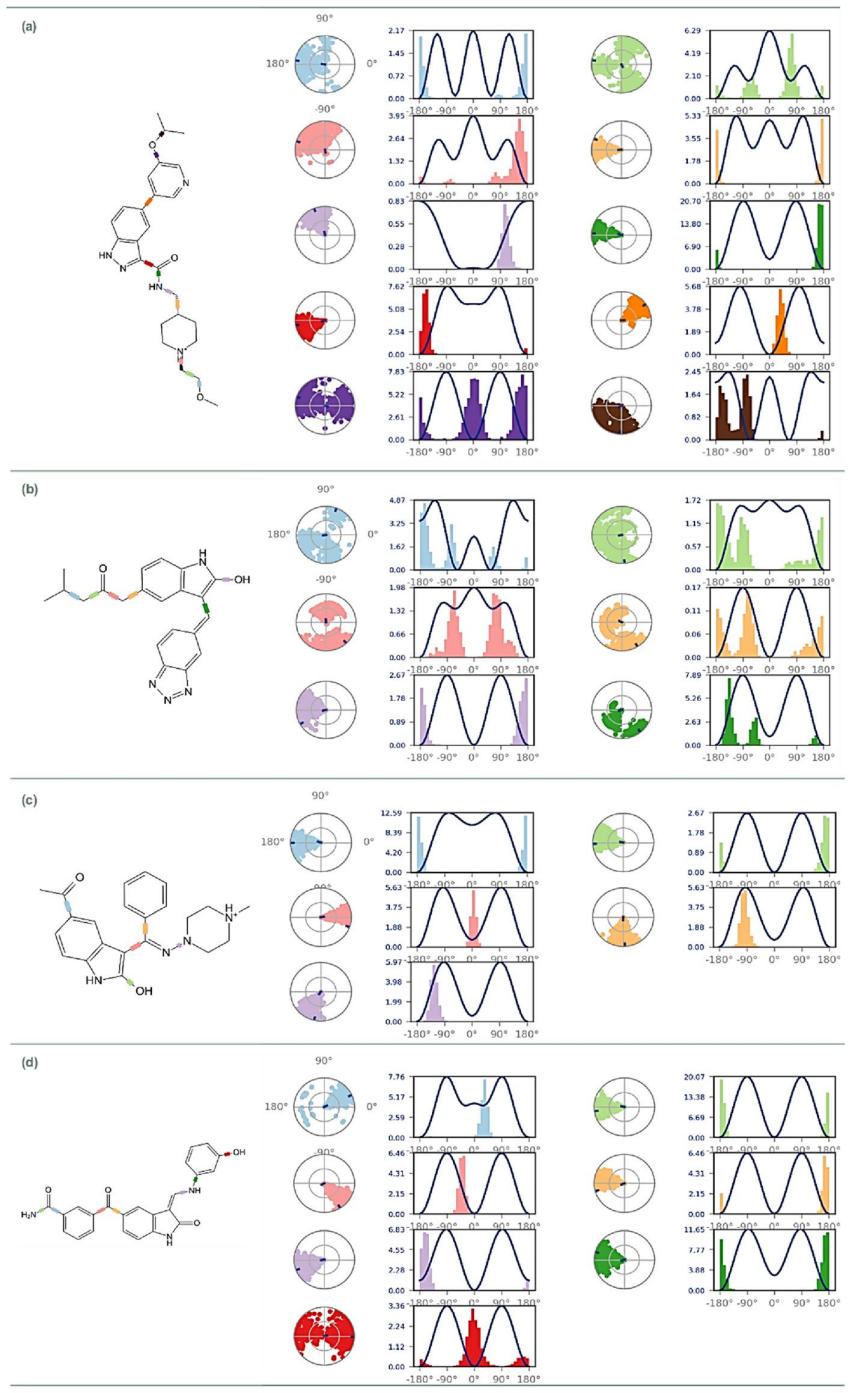
Ligand Torsion Profile for (**a**) OH8, (**b**) 1-[3-(benzotriazol-5-ylidenemethyl)-2-hydroxy-1H-indol-5-yl]-4-methylpentan-2-one (Rank 1), (**c**) 1-[2-hydroxy-3-[N-(4-methylpiperazin-1-yl)-C-phenylcarbonimidoyl]-1H-indol-5-yl]ethanone (Rank 2) and (**d**) 3-[2-hydroxy-3-[(3-hydroxyphenyl)iminomethyl]-1H-indole-5-carbonyl]benzamide (Rank 3) with their interaction with GSK3β (PDB ID: 6Y9R) during 100 ns MD simulation run.

Figure 10 provides a detailed analysis of the Ligand Torsion Profile during the 100 ns Molecular Dynamics (MD) simulation of interactions between GSK2β and OH8 (Fig. 10a), Rank 1 indirubin derivative (Fig. 10b), Rank 2 indirubin derivative (Fig. 10c), and Rank 3 indirubin derivative (Fig. 10d). The encompassed torsion plot in Fig. 10 elucidates the conformational journey of each ligand’s rotatable bond (RB) over the simulation span. Each torsion is uniquely color-coded, with a corresponding 2D ligand schematic atop the figure. Within each Fig. 10 subsection, the synergy between radial plots and bar graphs reveals the ligand’s torsional evolution throughout the simulation. The radial plot’s center signifies the simulation’s onset, with the torsional evolution being depicted in an expanding radial fashion. Meanwhile, the bar graphs synthesize the radial plots’ data by displaying the torsion’s probability density. If torsional potential data is accessible, the combined potential from related torsions is illustrated. This metric, in kilocalories per mole (kcal/mol), is presented on the chart’s left Y-axis. Such data is pivotal as it sheds light on potential conformational strains the ligand might endure in its protein-bound state. Grasping these ligand conformation nuances during binding is paramount for pinpointing possible drug design hurdles. For example, pronounced torsional stress could indicate a ligand’s potential difficulties in upholding its preferred conformation within the protein, which could consequentially influence its inhibitory potency. In summation, Fig. 10’s Ligand Torsion Profiles enrich our understanding of the ligands’ dynamics within the GSK2β binding site throughout the MD simulation. This depth of insight refines our grasp on these compounds’ viability as GSK2β inhibitors. By delineating the ligands’ conformational dynamics over the simulation, we acquire a clearer picture of their adaptability and capability to retain a conducive conformation for effective GSK2β engagement.

**Figure 11:**
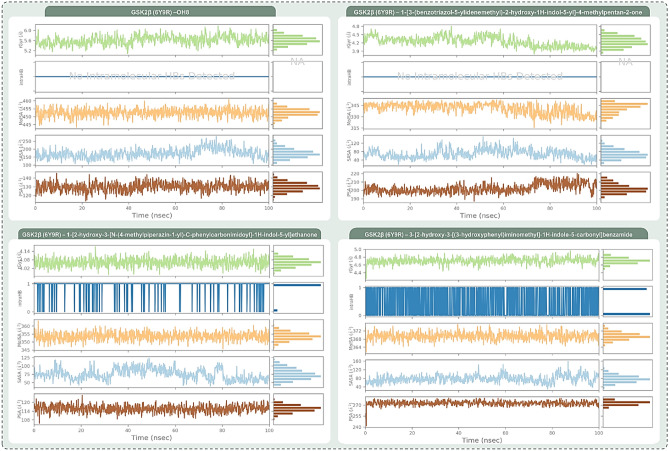
Ligand properties like Radius of Gyration (rGyr), intramolecular H bonds (intraHB), Molecular Surface Area (MolSA), Solvent-Accessible Surface Area (SASA) and Polar Surface Area (PSA) for (**a**) OH8, (**b**) 1-[3-(benzotriazol-5-ylidenemethyl)-2-hydroxy-1H-indol-5-yl]-4-methylpentan-2-one (Rank 1), (**c**) 1-[2-hydroxy-3-[N-(4-methylpiperazin-1-yl)-C-phenylcarbonimidoyl]-1H-indol-5-yl]ethanone (Rank 2) and (**d**) 3-[2-hydroxy-3-[(3-hydroxyphenyl)iminomethyl]-1H-indole-5-carbonyl]benzamide (Rank 3) with their interaction with GSK3β (PDB ID: 6Y9R) during 100 ns MD simulation run.

In next set of analysis, as showcased in Fig. 11, we immersed ourselves in examination of the ligand’s unique attributes, encompassing RMSD, radius of gyration (rGyr), intramolecular hydrogen bonds (intraHB), molecular surface area (MolSA), solvent accessible surface area (SASA), and polar surface area (PSA). The RMSD provides an insight into the structural stability and magnitude of ligand fluctuations during the simulation, which is crucial for gauging the simulation’s trajectory reliability. The rGyr, on the other hand, offers a measure of the ligand’s compactness, with a stable rGyr value indicating that the ligand maintains its structure throughout the simulation. Additionally, the intraHB, by monitoring the ligand’s internal hydrogen bonds, presents a snapshot of the ligand’s structural integrity, where a higher count typically underscores an increased rigidity. Turning our attention to MolSA, it offers a glimpse into the ligand’s overall shape and size, with any changes indicating potential conformational alterations during the simulation. Meanwhile, the SASA and PSA come forth as critical metrics for understanding the ligand’s interaction with its environment. While SASA provides an understanding of the ligand’s surface area exposed to solvents, indicating potential rearrangements or shifts in solvent interactions, the PSA plays a pivotal role in discerning the ligand’s solubility and permeability, often shedding light on its capability to traverse cell membranes or its solubility dynamics in the bloodstream. Taken together, these parameters present a comprehensive depiction of the ligand’s dynamic behavior during the simulation. They align with the core findings of the manuscript, providing an integral understanding that is essential for informed drug design initiatives.

In addition to the primary results depicted in Figs. 6, 7, 8, 9, 10 and 11, which illustrate the interaction profiles and stability metrics of the GSK2β-ligand complexes over a 100 ns MD simulation, supplementary analyses further corroborate these findings. The replicated versions of the MD simulations are detailed in Supplementary Figs. S1 to S6. These supplementary figures complement the primary data by providing additional perspectives on the interaction dynamics and stability of the GSK2β-ligand complexes, thus enriching the overall understanding of their dynamics.

**Figure 12:**
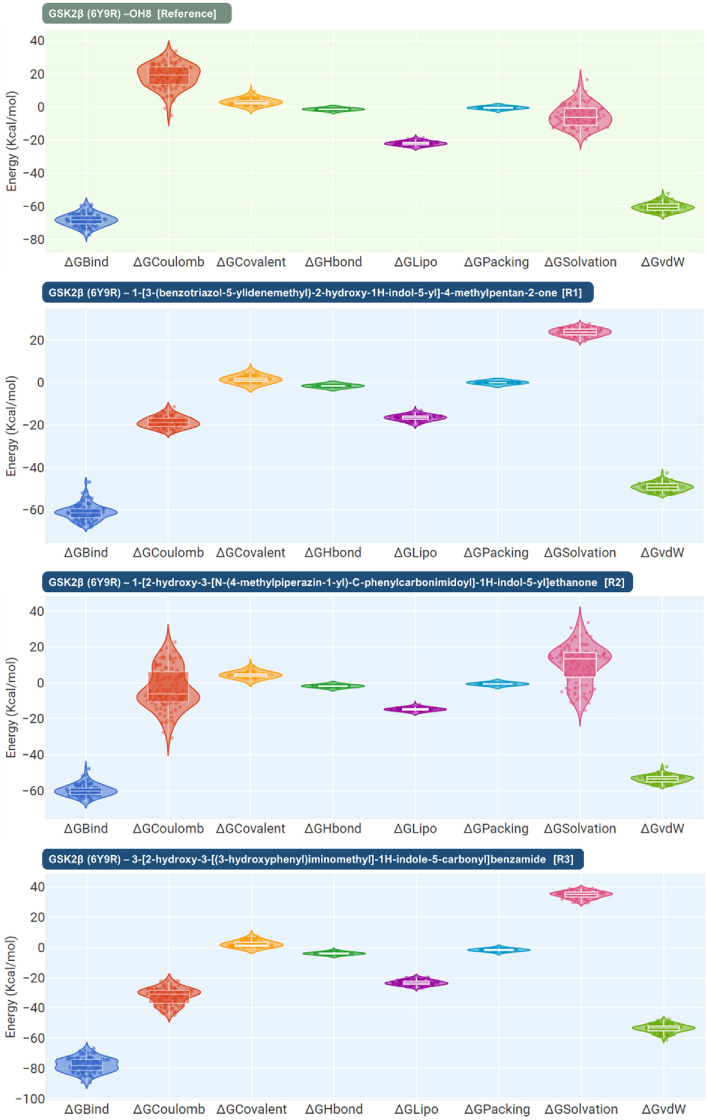
Post MD MM-GBSA assessment of OH8, 1-[3-(benzotriazol-5-ylidenemethyl)-2-hydroxy-1H-indol-5-yl]-4-methylpentan-2-one (Rank 1), 1-[2-hydroxy-3-[N-(4-methylpiperazin-1-yl)-C-phenylcarbonimidoyl]-1H-indol-5-yl]ethanone (Rank 2) and 3-[2-hydroxy-3-[(3-hydroxyphenyl)iminomethyl]-1H-indole-5-carbonyl]benzamide (Rank 3) in complex with GSK3β (PDB ID: 6Y9R), where ΔGBind = Binding energy, ΔGCoulomb = Coulomb energy, ΔGCovalent = Covalent bond energy, ΔGHbond = Hydrogen-bonding correction, ΔGLipo = Lipophilic energy, ΔGPacking = Pi-Pi packing correction, ΔGSolvation = Solvation energy and ΔGvdW = Van der Waals energy.

In Fig. 12, a meticulous MM-GBSA energy analysis is presented, capturing the binding dynamics between GSK2β (6Y9R) and a suite of inhibitory compounds, including the reference inhibitor OH8 and three other top-ranked candidates. This analysis is derived from 100 distinct snapshots taken from the last 10 ns (ns) of a 100 ns MD simulation, which collectively provide a robust picture of the ligand–protein interactions. The reference inhibitor OH8 sets a benchmark with an average ΔGBind of − 68.2250 kcal/mol, demonstrating a substantial binding affinity to GSK2β. The ΔGBind values fluctuate within a range from − 77.2297 to − 59.0284 kcal/mol, and a standard deviation of 3.57 kcal/mol, reflecting the dynamic and complex nature of molecular interactions.

Rank 1 compound shows a binding profile with an average ΔGBind of − 61.1383 kcal/mol, indicating a promising interaction that, while slightly less than OH8, remains within a competitive range. Its binding energy varies across a range from − 68.2032 to − 46.8695 kcal/mol, with a standard deviation of 3.70 kcal/mol, suggesting a diverse set of binding interactions. The Rank 2 compound exhibits a similar trend, with an average ΔGBind of − 59.9495 kcal/mol and a standard deviation of 3.05 kcal/mol, spanning a range from − 65.7829 to − 47.8731 kcal/mol. These values point to a consistent and reliable binding affinity, aligning closely with the reference inhibitor in terms of the binding energy profile. Rank 3 compound, while demonstrating a notable average ΔGBind of − 77.8837 kcal/mol, does not overshadow its counterparts. The range of its binding energies, from − 89.4061 to − 66.8994 kcal/mol, and a standard deviation of 4.72 kcal/mol, suggest strong and potentially favorable binding interactions, yet within the context of the dynamic ensemble presented by the other compounds.

The violin plots within Fig. 12 articulate the distribution of various energy components, such as electrostatic, covalent, hydrogen bonding, hydrophobic, packing, solvation, and Van der Waals energies, for each compound. These plots offer a granular view of how each energy component contributes to the overall binding affinity. Notably, the distributions across these energy components for the top-ranked compounds are comparable, with each showing strengths in different interaction types. The data depicted in Fig. 12 does not singularly favor any one of the top-ranked compounds; rather, it underscores their collective potential as GSK2β inhibitors. Each compound showcases a binding energy profile that is both dynamic and significant, highlighting the nuanced and multifaceted nature of their interactions with the target protein. The comprehensive energetic assessment suggests that all three compounds, alongside OH8, are potent contenders in the context of GSK2β inhibition and merit further exploration for therapeutic development.

### In-silico pharmacokinetics

The chemical properties of OH8 and Top 3 three Indirubin derivatives were compared to assess their drug-like properties (Table 2). Table 2 elucidates the chemical properties of the reference inhibitor OH8 and the top three Indirubin derivatives. The molecular weight of a compound can influence its pharmacokinetic profile, with larger molecules often having reduced cell permeability and potentially altered bioavailability. OH8, with a molecular weight of 451.571, is notably heavier than the top three Indirubin derivatives, which have molecular weights ranging from 360.417 to 399.406. The lipophilicity of a molecule, commonly represented by the LogP value, is a key parameter in drug design. It affects not only the drug’s absorption and distribution within the body but also its potential interactions with biological membranes and proteins. The LogP values of the reference inhibitor and the derivatives are in a close range, with OH8 having a value of 3.5003 and the derivatives varying between 3.0759 and 3.6596. This suggests that these compounds, including OH8, have somewhat similar lipophilic characteristics. Rotatable bonds in a molecule can influence its conformational flexibility, which in turn can impact its ability to interact dynamically with a protein target. OH8 has the highest number of rotatable bonds (9) compared to the derivatives, which have between 4 and 5. This indicates that OH8 might exhibit a higher conformational flexibility in comparison. The number of hydrogen bond acceptors and donors in a molecule can influence its interaction profile with proteins, as hydrogen bonds play a pivotal role in molecular recognition and binding. All compounds, including OH8, have a comparable number of acceptors, ranging from 5 to 6. However, the last Indirubin derivative stands out with 4 hydrogen bond donors, compared to 2 in the other compounds, which might lead to distinct interaction patterns. Lastly, the surface area of a molecule can provide insights into its size and shape, potentially affecting its interaction dynamics with the protein target. OH8 has the largest surface area at 194.224, while the derivatives exhibit slightly smaller values, ranging from 155.506 to 170.678. In summary, Table 2 provides a comprehensive overview of the physicochemical properties of OH8 and the top three Indirubin derivatives. While there are similarities in their profiles, subtle differences could lead to varied interaction dynamics and behaviors when these compounds interact with their respective protein targets.

**Table 2:** Chemical properties of reference inhibitor OH8 and top three Indirubin derivatives.

	OH8 (PubChem CID: 146036038)	1-[3-(benzotriazol-5-ylidenemethyl)-2-hydroxy-1H-indol-5-yl]-4-methylpentan-2-one (PubChem CID: 137047232)	1-[2-hydroxy-3-[N-(4-methylpiperazin-1-yl)-C-phenylcarbonimidoyl]-1H-indol-5-yl]ethenone (PubChem CID: 136618625)	3-[2-hydroxy-3-[(3-hydroxyphenyl)iminomethyl]-1H-indole-5-carbonyl]benzamide (PubChem CID: 136401024)
Structure				
Descriptor	Value	Value	Value	Value
Molecular Weight	451.571	360.417	376.46	399.406
LogP	3.5003	3.4919	3.0759	3.6596
#Rotatable Bonds	9	5	4	5
#Acceptors	6	5	5	5
#Donors	2	2	2	4
Surface Area	194.224	155.506	163.634	170.678

Table 3 offers a comprehensive exploration of the ADMET (Absorption, Distribution, Metabolism, Excretion, and Toxicity) properties of the reference inhibitor OH8 and the top three Indirubin derivatives. The properties listed encompass essential pharmacokinetic parameters that influence a drug’s efficacy, safety, and overall therapeutic potential. In terms of absorption, water solubility provides insights into the potential of a molecule to dissolve in aqueous solutions, influencing bioavailability. The values, ranging from − 3.261 (for OH8) to − 4.165, are provided in logarithmic units of mol/L. Caco2 permeability is a measure of how well a compound can permeate through intestinal cells, with OH8 having a value of 0.462, whereas the top three Indirubin derivatives exhibit a range from − 0.294 to 1.052, indicating their varying permeability potentials. Another critical aspect of absorption is intestinal absorption, where all the compounds show percentages from 78.286 to 94.867%, suggesting good absorption potential in humans. Moreover, all the compounds are predicted as substrates and inhibitors for P-glycoprotein, a crucial efflux pump which can impact drug absorption and distribution. The distribution parameters underscore the potential of these compounds to spread throughout the body. The VDss represents the volume of distribution, indicating how extensively a drug disperses away from the bloodstream. OH8 has a VDss value of 2.02, while the derivatives vary between − 0.442 and 1.48. The fraction unbound reveals the portion of the drug that remains free in the bloodstream, with OH8 having a fraction of 0.177. Metabolism, a vital phase in drug pharmacokinetics, determines how the body processes and breaks down drugs. All compounds, including OH8, are predicted substrates for CYP3A4, a major enzyme responsible for drug metabolism in the liver. However, only some of the derivatives inhibit other cytochrome P450 enzymes, which can have implications for drug-drug interactions. Excretion properties, like total clearance, provide insights into the rate at which these compounds are removed from the body. OH8 has a clearance value of 0.91, with the derivatives ranging from 0.279 to 0.636. Toxicity parameters are paramount in drug development, shedding light on potential adverse effects. OH8, for example, is not predicted to be toxic in the AMES test, whereas two of the derivatives are. The maximum tolerated dose provides insights into the highest dose that can be administered without causing adverse effects, and these values vary across the compounds. Other toxicity measures, such as hepatotoxicity and skin sensitization, further emphasize the safety profiles of these compounds. By understanding these parameters, researchers can better gauge the therapeutic promise of these compounds and their potential roles in drug development.

**Table 3:** ADMET properties of reference inhibitor OH8 and top three Indirubin derivatives.

Property	Model name	Predicted Values of Ligand				Unit
		OH8 (PubChem CID: 146036038)	1-[3-(benzotriazol-5-ylidenemethyl)-2-hydroxy-1H-indol-5-yl]-4-methylpentan-2-one (PubChem CID: 137047232)	1-[2-hydroxy-3-[N-(4-methylpiperazin-1-yl)-C-phenylcarbonimidoyl]-1H-indol-5-yl]ethenone (PubChem CID: 136618625)	3-[2-hydroxy-3-[(3-hydroxyphenyl)iminomethyl]-1H-indole-5-carbonyl]benzamide (PubChem CID: 136401024)	
Absorption	Water solubility	− 3.261	− 4.165	− 3.6	− 3.52	Numeric (log mol/L)
Absorption	Caco2 permeability	0.462	1.024	1.052	− 0.294	Numeric (log Papp in 10^−6^ cm/s)
Absorption	Intestinal absorption (human)	78.286	94.867	92.771	81.265	Numeric (% Absorbed)
Absorption	Skin Permeability	− 2.735	− 2.738	− 2.81	− 2.735	Numeric (log Kp)
Absorption	P-glycoprotein substrate	Yes	Yes	Yes	Yes	Categorical (Yes/No)
Absorption	P-glycoprotein I inhibitor	Yes	Yes	Yes	Yes	Categorical (Yes/No)
Absorption	P-glycoprotein II inhibitor	Yes	Yes	Yes	Yes	Categorical (Yes/No)
Distribution	VDss (human)	2.02	0.064	1.48	− 0.442	Numeric (log L/kg)
Distribution	Fraction unbound (human)	0.177	0	0.132	0	Numeric (Fu)
Distribution	BBB permeability	− 1.375	− 0.242	− 0.477	− 0.838	Numeric (log BB)
Distribution	CNS permeability	− 2.961	− 2.247	− 2.197	− 2.316	Numeric (log PS)
Metabolism	CYP2D6 substrate	No	No	No	No	Categorical (Yes/No)
Metabolism	CYP3A4 substrate	Yes	Yes	Yes	Yes	Categorical (Yes/No)
Metabolism	CYP1A2 inhibitor	No	Yes	Yes	Yes	Categorical (Yes/No)
Metabolism	CYP2C19 inhibitor	No	Yes	No	Yes	Categorical (Yes/No)
Metabolism	CYP2C9 inhibitor	No	Yes	No	Yes	Categorical (Yes/No)
Metabolism	CYP2D6 inhibitor	No	No	No	No	Categorical (Yes/No)
Metabolism	CYP3A4 inhibitor	Yes	Yes	Yes	Yes	Categorical (Yes/No)
Excretion	Total Clearance	0.91	0.332	0.636	0.279	Numeric (log ml/min/kg)
Excretion	Renal OCT2 substrate	Yes	No	No	No	Categorical (Yes/No)
Toxicity	AMES toxicity	No	Yes	No	Yes	Categorical (Yes/No)
Toxicity	Max. tolerated dose (human)	0.334	− 0.196	− 0.381	0.394	Numeric (log mg/kg/day)
Toxicity	hERG I inhibitor	No	No	No	No	Categorical (Yes/No)
Toxicity	hERG II inhibitor	Yes	Yes	Yes	Yes	Categorical (Yes/No)
Toxicity	Oral Rat Acute Toxicity (LD50)	2.717	2.446	2.774	2.667	Numeric (mol/kg)
Toxicity	Oral Rat Chronic Toxicity (LOAEL)	1.816	1.579	1.613	2.566	Numeric (log mg/kg_bw/day)
Toxicity	Hepatotoxicity	Yes	No	Yes	Yes	Categorical (Yes/No)
Toxicity	Skin Sensitization	No	No	No	No	Categorical (Yes/No)
Toxicity	*T. pyriformis* toxicity	0.305	0.406	0.529	0.293	Numeric (log ug/L)
Toxicity	Minnow toxicity	0.128	0.056	2.098	1.573	Numeric (log mM)

## Discussion

Indirubin derivatives, with their origin in the natural pigment indirubin, exhibit a broad spectrum of biological activities such as anti-inflammatory, anti-cancer, and neuroprotective effects, underscoring their significant therapeutic potential. These compounds have the unique ability to modulate multiple disease-related signaling pathways, such as NF-κB, STAT3, and Wnt, positioning them as potential game-changers in disease treatment^6,8,9^. Particularly, their capability to address challenges associated with conventional chemotherapy, such as overcoming multidrug resistance and selective targeting of cancer cells while sparing healthy ones, underscores their potential as innovative drugs. Although they've exhibited efficacy against a plethora of cancers and other conditions like diabetes and psoriasis, further research is required to optimize their pharmacological properties and ensure their safe and effective translation into clinical practice^6,8–10^. The versatility and potency of indirubin derivatives underscore their potential as the next generation of therapeutic agents.

In our study, we conducted a thorough computational analysis to elucidate the molecular interaction dynamics of indirubin derivatives with GSK3β, unraveling the core mechanisms at play. Three compounds, namely 1-[3-(benzotriazol-5-ylidenemethyl)-2-hydroxy-1H-indol-5-yl]-4-methylpentan-2-one (Rank 1, PubChem CID: 137047232), 1-[2-hydroxy-3-[N-(4-methylpiperazin-1-yl)-C-phenylcarbonimidoyl]-1H-indol-5-yl]ethanone (Rank 2, CID: 136618625), and 3-[2-hydroxy-3-[(3-hydroxyphenyl)iminomethyl]-1H-indole-5-carbonyl]benzamide (Rank 3, CID: 136401024), stood out due to their robust inhibitory activity against GSK3β combined with their promising ADMET profiles, underscoring their potential as effective drug candidates. Our results align with previous studies that spotlight the notable anticancer effectiveness and pharmacokinetic merits of these derivatives. While these molecules have been previously tested on various cell lines and have demonstrated interactions with proteins in the GSK3 family, in-depth computational studies detailing these interactions have been sparse. Our research seeks to address this knowledge gap by providing an intricate computational perspective on these interactions, thereby enriching the scientific understanding and setting the stage for further rigorous investigations. Such a nuanced exploration augments the understanding of these compounds’ potential in a broader and more integrated context.

The compound of prime focus in our study, termed Rank 1, is 1-[3-(benzotriazol-5-ylidenemethyl)-2-hydroxy-1H-indol-5-yl]-4-methylpentan-2-one (PubChem CID: 137047232). The importance of this compound is underscored by its inclusion in two patents, both titled "Indazolyl, benzimidazolyl, benzotriazolyl substituted indolinone derivatives as kinase inhibitors useful in the treatment of cancer". The earlier patent, US-2011065702-A1^38^, and the later one, US-8765748-B2^39^, represent critical documents that establish the compound’s importance in cancer treatment research. The patents elucidate a spectrum of indolinone compounds that function as potent kinase inhibitors, predominantly targeting polo-like kinases (PLK) and Aurora B. The structural composition of these compounds adheres to a specific formula (A). In this formula, the different ring structures and their respective substituents play crucial roles in determining the compound’s potency and specificity. Notably, the patented compounds have not only been described in terms of their structure but have also been accompanied by a detailed exposition on their synthesis, biological activity, and potential pharmaceutical formulations. The utilization of these compounds, particularly Rank 1, for cancer treatment and the inhibition of Aurora B and/or PLK-4 manifests as a primary claim in the patents. This sets a significant backdrop to our study and its findings. At this juncture, a pertinent question arises: given the extant knowledge and patent protection of these compounds, why is our study indispensable? One might argue that the in vitro validation of these compounds should be the next logical step. However, this becomes redundant as these compounds have already been tested on an array of cell lines and their efficacy underscored in the patents. The crux of our study lies not in the mere identification of these compounds as potential inhibitors but in the nuanced, comparative assessment we provide. While there might be an abundance of patented compounds with semblances to those in our manuscript, our computational approach discerningly sifts through this plethora to pinpoint the most potent inhibitor for the protein GSK3β. This is paramount, given the limited published information that offers such a comparative evaluation of multiple compounds within a singular research framework. In essence, our study doesn’t merely reiterate what is known; it elevates the knowledge by bridging gaps, offering comparative insights, and reinforcing the potential of Rank 1 and its analogs in therapeutic interventions.

The compound 1-[2-hydroxy-3-[N-(4-methylpiperazin-1-yl)-C-phenylcarbonimidoyl]-1H-indol-5-yl]ethanone, designated as Rank 2 (CID: 136618625), holds a position of prominence within two patents. The first, titled "Novel cycloalkyl-containing 5-acylindolinones, their preparation and their use as pharmaceutical products", is identified by the Patent EP-1727812-B1^40^. This patent delineates the innovative cycloalkyl-containing 5-acylindolinones with their structural orientation aligning with the general formula (I). The spotlight is on their synthesis and potential pharmaceutical applications. A hallmark of these compounds is their pharmacological prowess, most notably manifesting as inhibitors of protein kinases, with a distinct affinity towards GSK3. The patent meticulously outlines synthesis routes for these compounds, leveraging 2-indolinone derivatives in conjunction with varied amines. Additionally, it sheds light on the biological activities of these compounds, both in vitro and in vivo. The subsequent patent, recognized by the publication number ES-2320564-T3, is titled “New 5-Acilindolinonas With Content In Cyclalkyl, Its Preparation And Its Use As Medications”^41^. This patent converges on similar themes, emphasizing the cycloalkyl-rich 5-acylindolinones. A remarkable observation from this patent is the potency of indirubin derivatives as formidable inhibitors of GSK3β, boasting IC values oscillating between 5 and 50 nM. Beyond the mere structural and synthetic discussion, this patent broaches the compounds’ therapeutic potential. It proposes their efficacy against a myriad of conditions tied to aberrant GSK3 activity. This spans from diabetes mellitus (both types I and II) to neurotraumatic injuries, from neurodegenerative maladies to bipolar disorders. In synthesizing the insights from both patents, Rank 2 emerges as a compound of considerable potential. Its pharmacological attributes, especially against GSK3β, coupled with its therapeutic promise against a range of diseases, underscore its clinical relevance. Furthermore, its mention in multiple patents amplifies its significance, situating it as a lead candidate in the quest for efficacious kinase inhibitors. The depth of research into its synthesis, biological activity, and therapeutic applications converges to highlight the pivotal role Rank 2 could play in future pharmacological interventions.

The compound 3-[2-hydroxy-3-[(3-hydroxyphenyl)iminomethyl]-1H-indole-5-carbonyl]benzamide, designated as Rank 3 (CID: 136401024), features prominently in two patents, each underscoring its therapeutic importance. The first patent, identified by the publication number WO-2010075197-A1^42^, delves deep into the realm of drug delivery systems, especially ocular implants. Central to these implants are indirubin derivatives that act as tyrosine kinase inhibitors (TKIs). A ground-breaking revelation from this patent is the implant’s capability to release TKIs in a methodical, sustained fashion into the eye, targeting ocular ailments rooted in abnormal tyrosine kinase signal transduction. This includes afflictions such as macular degeneration, diabetic retinopathy, and glaucoma. The patent accentuates the potency of indirubin derivatives in inhibiting a spectrum of tyrosine kinases like CDKs, GSK3β, and JNKs – all pivotal in cellular signalling pathways. By impeding these kinases, indirubin derivatives possess the potential to rectify or halt pathological processes, thereby ameliorating the ocular conditions. Complementing these insights, the patent elucidates the synthesis methods of these ocular implants and showcases the efficacy and safety profile based on animal model studies. The subsequent patent, recognized by the publication number US-2007032478-A1^43^, pivots around kinase inhibitors. These compounds, vital in modulating cellular pathways like growth, division, and signalling, have profound implications in the onset and progression of diseases like cancer and inflammation. The patent introduces a series of indirubin derivatives, emphasizing their anti-cancer and anti-inflammatory properties by virtue of kinase inhibition. A standout feature of this patent is its claim on certain indirubin derivatives exhibiting heightened selectivity and potency against GSK3—a kinase instrumental in cellular functions and implicated in diseases like Alzheimer’s. The patent furnishes detailed information on the chemical structures, synthesis pathways, and biological activities of these indirubin derivatives. Synthesizing insights from both patents, Rank 3 emerges not merely as a compound of note but as a beacon of therapeutic promise. Its association with ocular drug delivery and broad-spectrum kinase inhibition positions it as a pivotal player in future medical interventions. Its presence in multiple patents further cements its significance, offering a promising roadmap for drug discovery and development in the domains of ocular health and kinase-linked disorders.

Modern drug discovery greatly benefits from computational studies like molecular docking, MD simulations, MM-GBSA, and ADMET analysis. These digital techniques illuminate how potential drugs interact with target proteins, their binding strengths, the dynamics of these interactions, and their overall drug-related properties, enhancing drug design optimization^44–47^. With this in mind, we theorize that a thorough computational examination of cyclin GSK3β could shed light on the interaction intricacies of indirubin and indigo derivatives, enhancing existing anticancer activity data. However, our ADMET analysis did pinpoint some inconsistencies, especially concerning hepatotoxicity and P-glycoprotein inhibitor activity^48^. These could stem from the inherent limitations of the computational models employed. Such models might overlook intricate molecular interactions, metabolic pathways, and other pharmacokinetic factors. Moreover, real-life biological complexities might not always align with computational predictions. For instance, several variables, including dosage and individual susceptibilities, might influence a compound’s hepatotoxicity^49^. A compound’s activity with P-glycoprotein might also vary based on numerous parameters. While these in silico analyses offer preliminary insights, they warrant further validation through laboratory and clinical tests. Despite these challenges, our research underscores the promise of these compounds as GSK3β inhibitors. The top candidates from our study displayed strong and consistent interactions with GSK3β, showing promise as drugs. Our work not only reinforces the potential of indirubin derivatives but also aids in understanding the molecular dynamics involved. Such insights are pivotal for the future development of more potent GSK3β inhibitors.

### Supplementary Information

Supplementary Figures.

## Data Availability

All data generated or analyzed during this study are included in this published article.
