# Supplementary material for: Pinpointing top inhibitors for GSK3β from pool of indirubin derivatives using rigorous computational workflow and their validation using molecular dynamics (MD) simulations
Source: Sci Rep. 2024 Jan 2;14:49. doi: 10.1038/s41598-023-50992-7 (PMC10761884; doi:10.1038/s41598-023-50992-7)
Supplement: Supplementary file 1 — Supplementary Figures. [file 41598_2023_50992_MOESM1_ESM.docx]

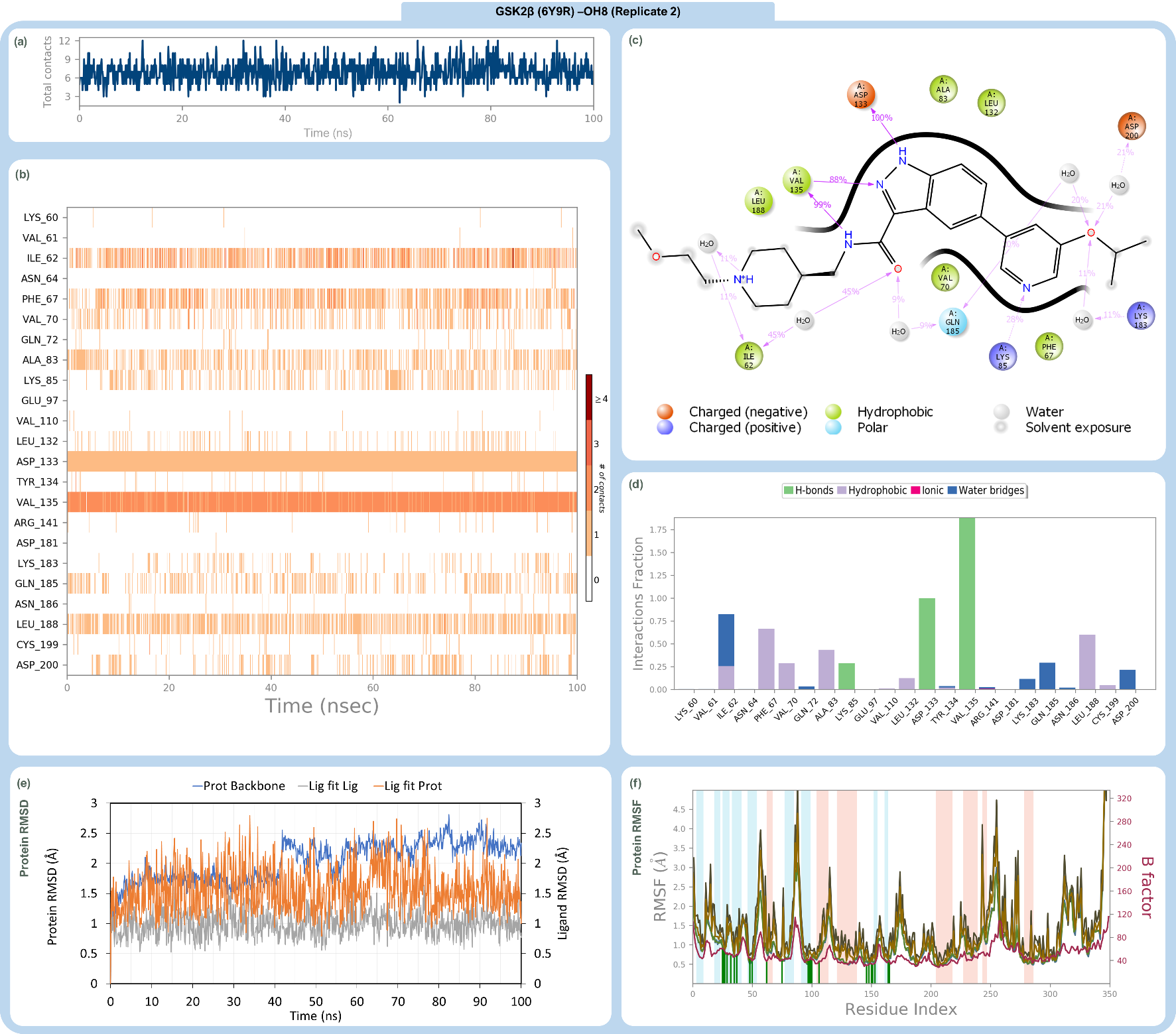


**Figure S1** Post MD simulation assessment showing (a) total contacts, (b) amino acid interaction timeline, (c) percent interaction profile and (d) interaction fraction profile, (e) Protein-Ligand RMSD and (f) Protein RMSF of GSK3β (PDB ID: 6Y9R) – OH8 (Reference inhibitor) complex.


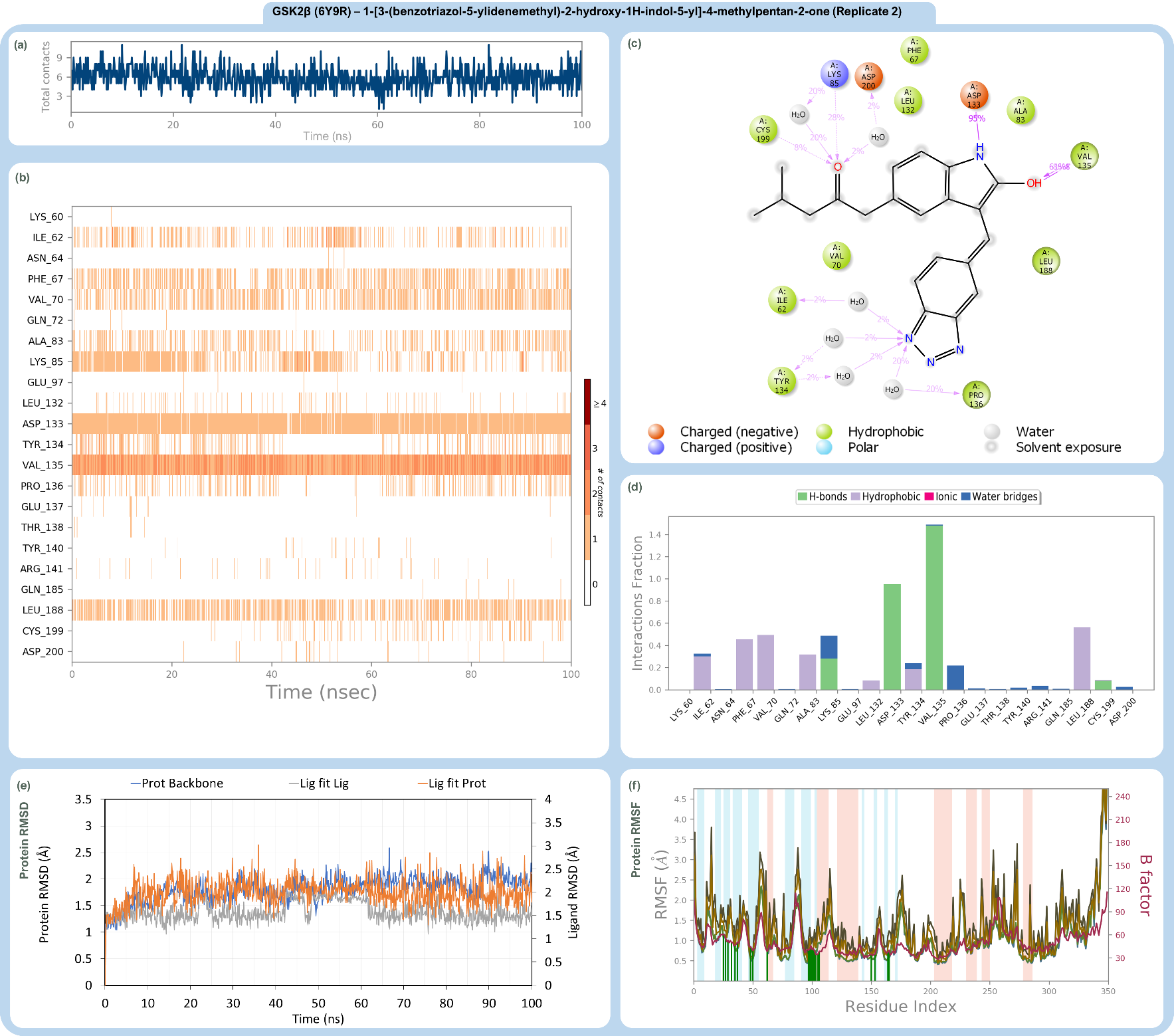


**Figure S2** Post MD simulation assessment showing (a) total contacts, (b) amino acid interaction timeline, (c) percent interaction profile and (d) interaction fraction profile, (e) Protein-Ligand RMSD and (f) Protein RMSF of GSK3β (PDB ID: 6Y9R) – 1-[3-(benzotriazol-5-ylidenemethyl)-2-hydroxy-1H-indol-5-yl]-4-methylpentan-2-one (Rank 1) complex.

**
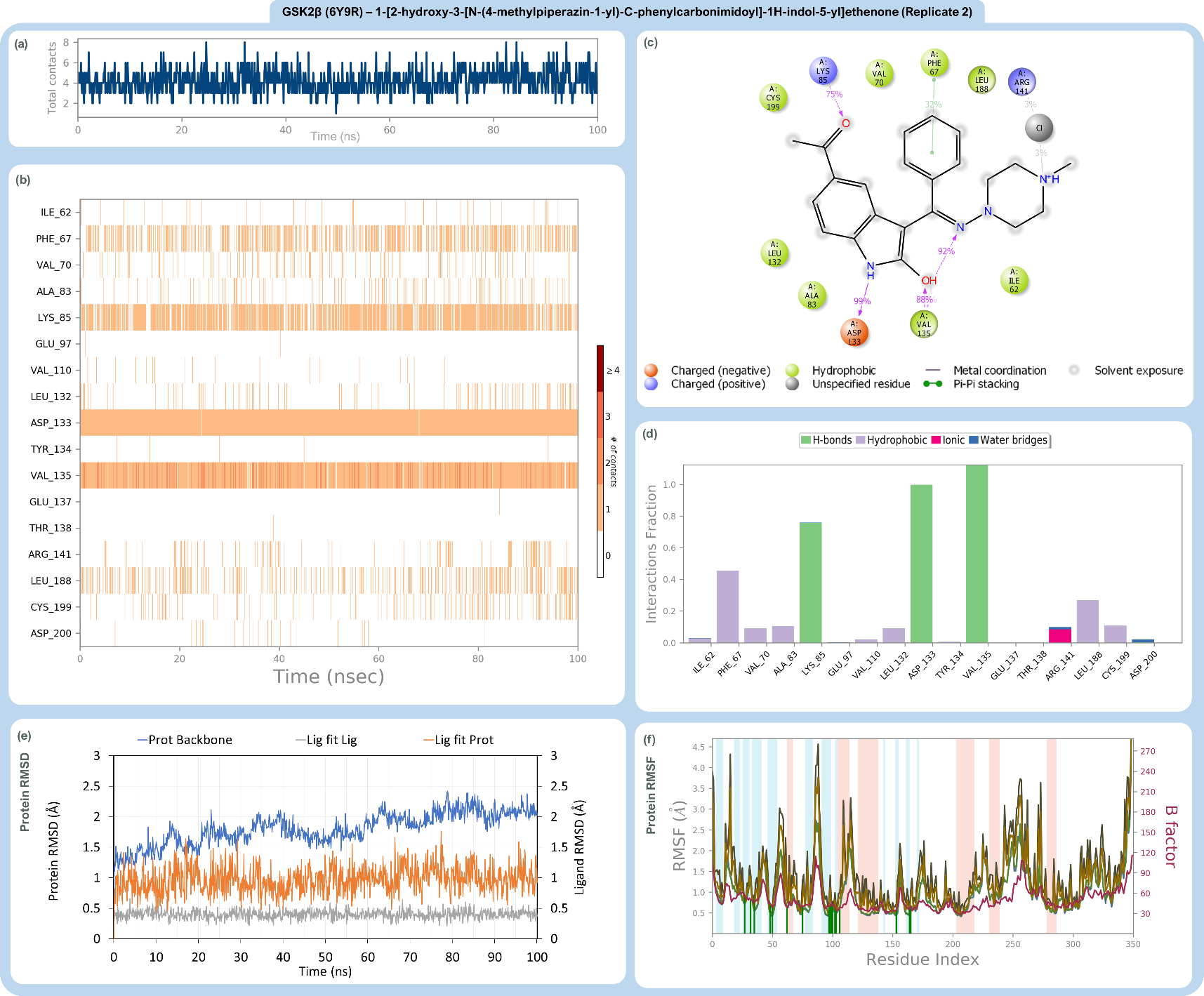
**

**Figure S3** Post MD simulation assessment showing (a) total contacts, (b) amino acid interaction timeline, (c) percent interaction profile and (d) interaction fraction profile, (e) Protein-Ligand RMSD and (f) Protein RMSF of GSK3β (PDB ID: 6Y9R) – 1-[2-hydroxy-3-[N-(4-methylpiperazin-1-yl)-C-phenylcarbonimidoyl]-1H-indol-5-yl]ethanone (Rank 2) complex.


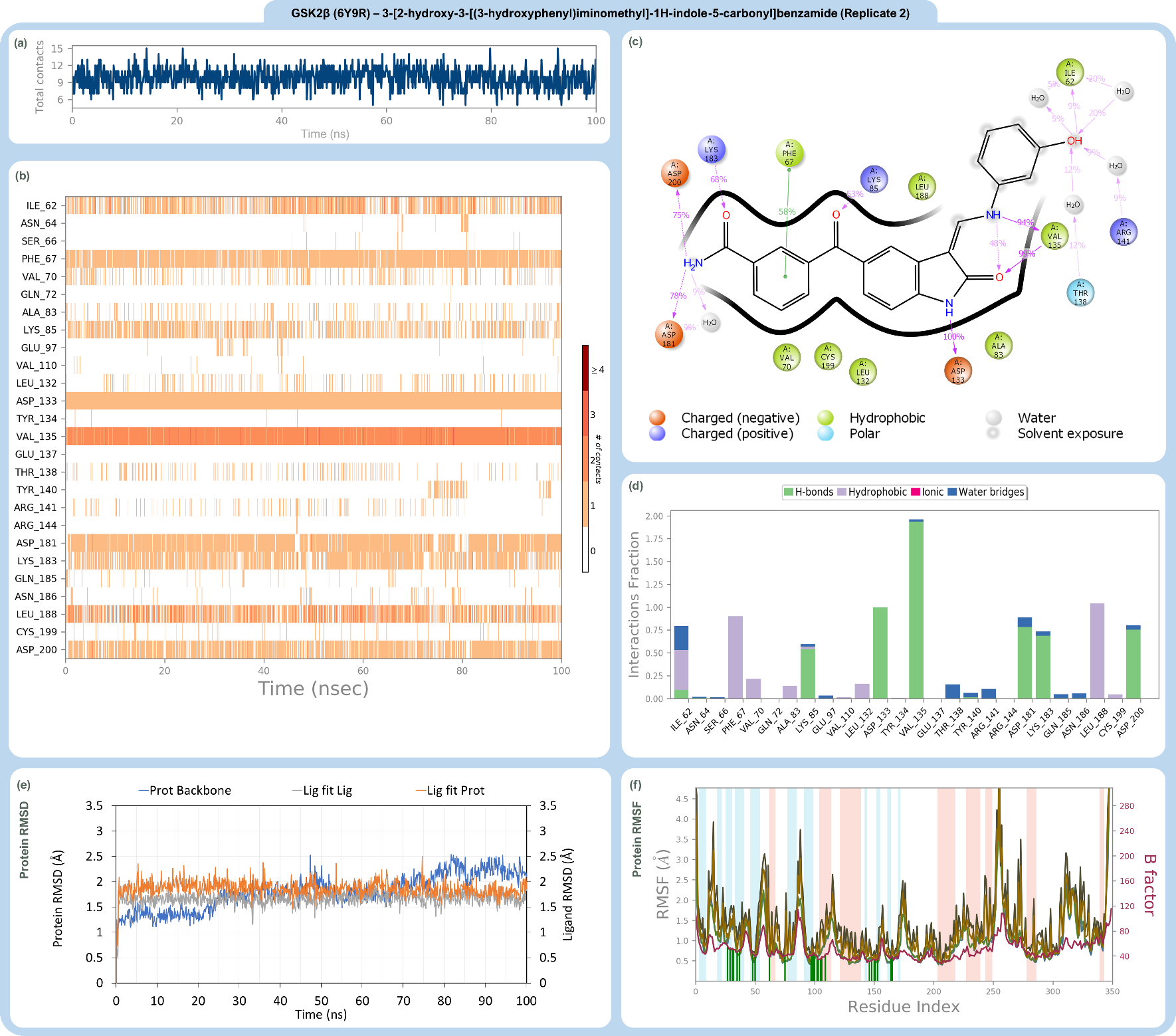


**Figure S4** Post MD simulation assessment showing (a) total contacts, (b) amino acid interaction timeline, (c) percent interaction profile and (d) interaction fraction profile, (e) Protein-Ligand RMSD and (f) Protein RMSF of GSK3β (PDB ID: 6Y9R) – 3-[2-hydroxy-3-[(3-hydroxyphenyl)iminomethyl]-1H-indole-5-carbonyl]benzamide (Rank 3) complex.


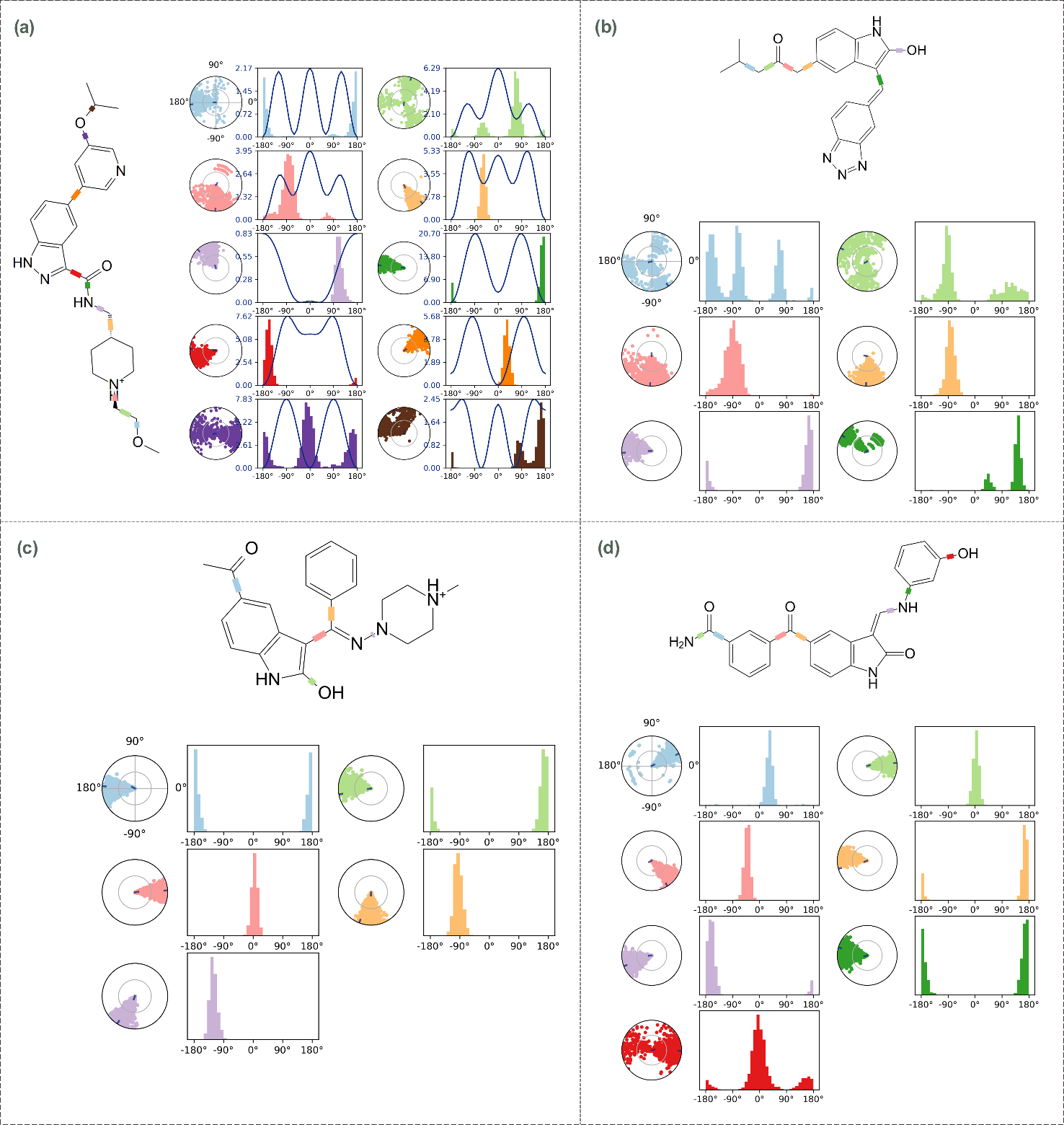


**Figure S5** Ligand Torsion Profile for **(a)** OH8, **(b)** 1-[3-(benzotriazol-5-ylidenemethyl)-2-hydroxy-1H-indol-5-yl]-4-methylpentan-2-one (Rank 1), **(c)** 1-[2-hydroxy-3-[N-(4-methylpiperazin-1-yl)-C-phenylcarbonimidoyl]-1H-indol-5-yl]ethanone (Rank 2) and **(d)** 3-[2-hydroxy-3-[(3-hydroxyphenyl)iminomethyl]-1H-indole-5-carbonyl]benzamide (Rank 3) with their interaction with GSK3β (PDB ID: 6Y9R) during 100 ns MD simulation run.


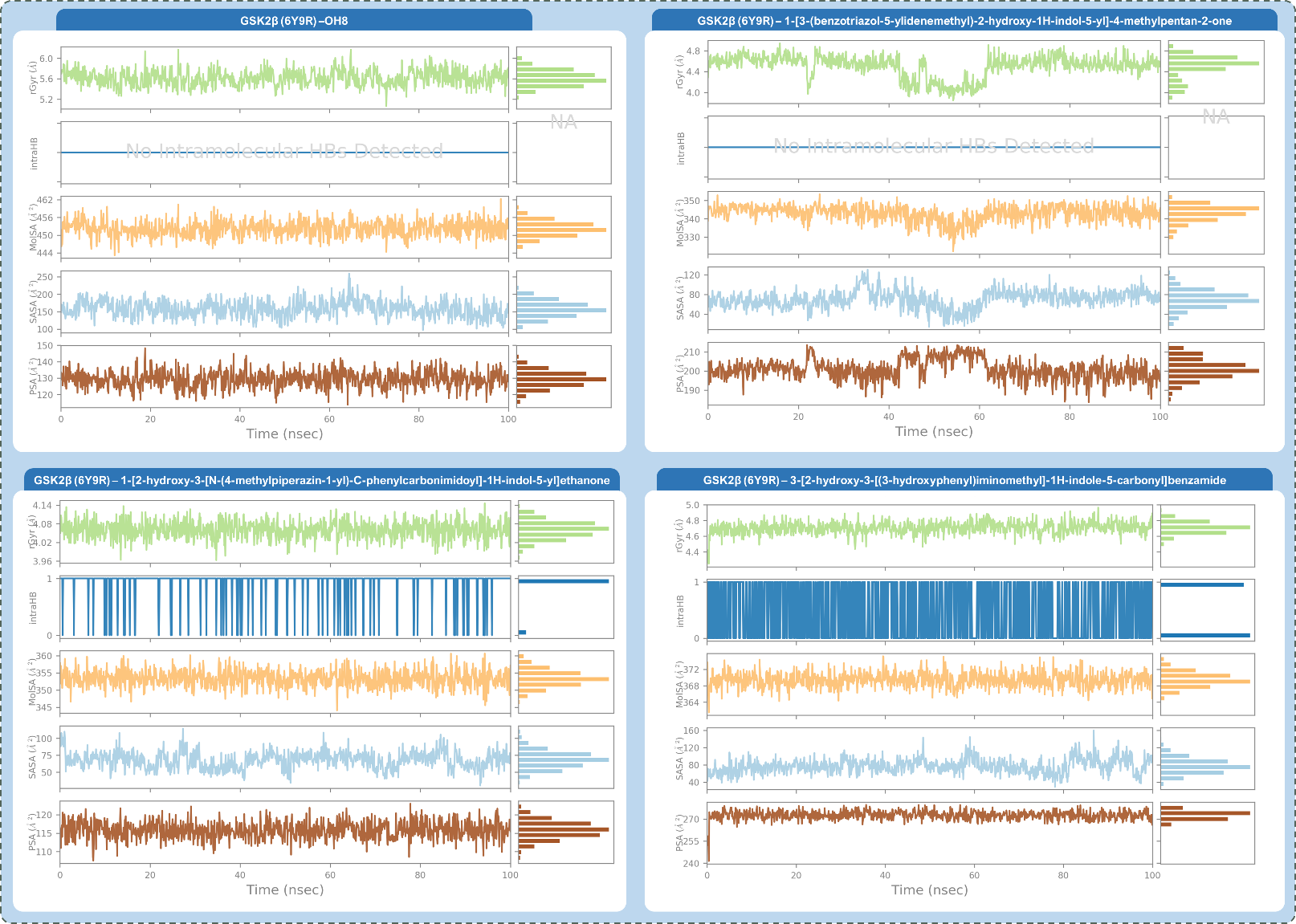


**Figure S6** Ligand properties like Radius of Gyration (rGyr), intramolecular H bonds (intraHB), Molecular Surface Area (MolSA), Solvent-Accessible Surface Area (SASA) and Polar Surface Area (PSA) for **(a)** OH8, **(b)** 1-[3-(benzotriazol-5-ylidenemethyl)-2-hydroxy-1H-indol-5-yl]-4-methylpentan-2-one (Rank 1), **(c)** 1-[2-hydroxy-3-[N-(4-methylpiperazin-1-yl)-C-phenylcarbonimidoyl]-1H-indol-5-yl]ethanone (Rank 2) and **(d)** 3-[2-hydroxy-3-[(3-hydroxyphenyl)iminomethyl]-1H-indole-5-carbonyl]benzamide (Rank 3) with their interaction with GSK3β (PDB ID: 6Y9R) during 100 ns MD simulation run.
